# Design, synthesis and antiproliferative screening of newly synthesized acrylate derivatives as potential anticancer agents†

**DOI:** 10.1039/d3ra03849a

**Published:** 2023-08-04

**Authors:** Dalal Sulaiman Alshaya, Rana M. O. Tawakul, Islam Zaki, Ali H. Abu Almaaty, Eman Fayad, Yasmin M. Abd El-Aziz

**Affiliations:** a Department of Biology, College of Science, Princess Nourah bint Abdulrahman University P.O. Box 84428 Riyadh 11671 Saudi Arabia; b Zoology Department, Faculty of Science, Port Said University Port Said 42526 Egypt; c Pharmaceutical Organic Chemistry Department, Faculty of Pharmacy, Port Said University Port Said 42526 Egypt Eslam.Zaki@pharm.psu.edu.eg; d Department of Biotechnology, Faculty of Sciences, Taif University P.O. Box 11099 Taif 21944 Saudi Arabia

## Abstract

A new series of acrylic acid and acrylate ester derivatives as modified analogs of tubulin polymerization inhibitors were designed and synthesized. The antiproliferative activity of the constructed molecules was investigated against MCF-7 breast carcinoma cells using CA-4 as positive molecule. Methyl acrylate ester 6e emerged as the most potent cytotoxic agent against MCF-7 cells, with an IC_50_ value of 2.57 ± 0.16 μM. Also, methyl acrylate ester molecule 6e showed good β-tubulin polymerization inhibition activity. Cellular cycle analysis showed that compound 6e can arrest MCF-7 cells at the G2/M phase. In addition, this compound produced a significant increase in apoptotic power as compared to control untreated MCF-7 cells. Furthermore, the effect of acrylate ester 6e on the gene expression levels of p53, Bax and Bcl-2 was investigated. This molecule increased the expression levels of both p53 and Bax, and decreased the gene expression level of Bcl-2 as compared to control untreated MCF-7 carcinoma cells.

## Abstract 2

A sequence of novel acrylic acids and acrylic esters has been synthesized and evaluated for *in vitro* cytotoxicity against MCF-7 breast cancer cells.

## Graphical abstract

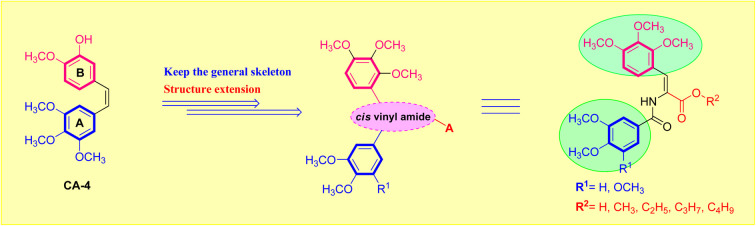

## 1. Introduction

The importance of microtubule polymerization in cancer control has been emphasized.^1,2^ Microtubule formation, comprising α/β tubulin heterodimers, has a crucial role in cellular processes such as maintaining cellular shape and cellular division of eukaryotic cells and is therefore regarded as an outstanding molecular target for chemotherapy.^3–5^ Tubulin polymerization is required for microtubule formation.^6^ Tubulin assembly inhibitors interfere with the tubulin-microtubule polymerization–depolymerization process and are becoming an attractive strategy for the development of highly efficient anticancer drugs.^7–9^ Several natural products, such as colchicine, paclitaxel, and the vinca alkaloids, inhibit tubulin polymerization by binding to tubulin at their respective binding sites.^10,11^ In the case of tubulin polymerization inhibition at the colchicine binding site, combretastatin A-4 (CA-4) is a *cis*-stilbenoid molecule that elicited remarkable β-tubulin polymerization suppression activity acting at the colchicine site.^12–14^ CA-4 is the lead antimitotic molecule within the combretastatin family which exerts outstanding antitumor activity on various cancer cells due to β-tubulin polymerization suppression activity and anti-vascular effect.^15,16^ This is in addition to its potency against multidrug resistant cancer cell line.^17^ A property that makes analogs of CA-4 attractive for further development of more appropriate anticancer molecules which could be utilized in clinical use to overcome the drawbacks of conventional anticancer regimens, especially development of drug resistance.^18–20^

Acrylate moiety is a common structural scaffold in the structure of numerous natural and synthetic small compounds displaying versatile biological interests.^21,22^ It has been reported to exhibit diverse biomedical activities, including anticancer activity.^23^ Their development has introduced new molecules acting as anticancer agents through different mechanisms such as β-tubulin inhibition and protein kinase inhibition.^24,25^ These findings suggested that acrylate pharmacophore is a promising molecular scaffold for further modification to develop more effective anticancer drug candidates.^26^

**Fig. 1:**
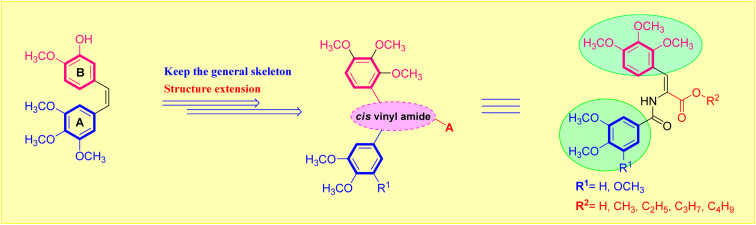
Designed strategy of the target acrylate derivatives 5a,b and 6a–i. A = carboxylic acid or ester group.

**Fig. 2:**
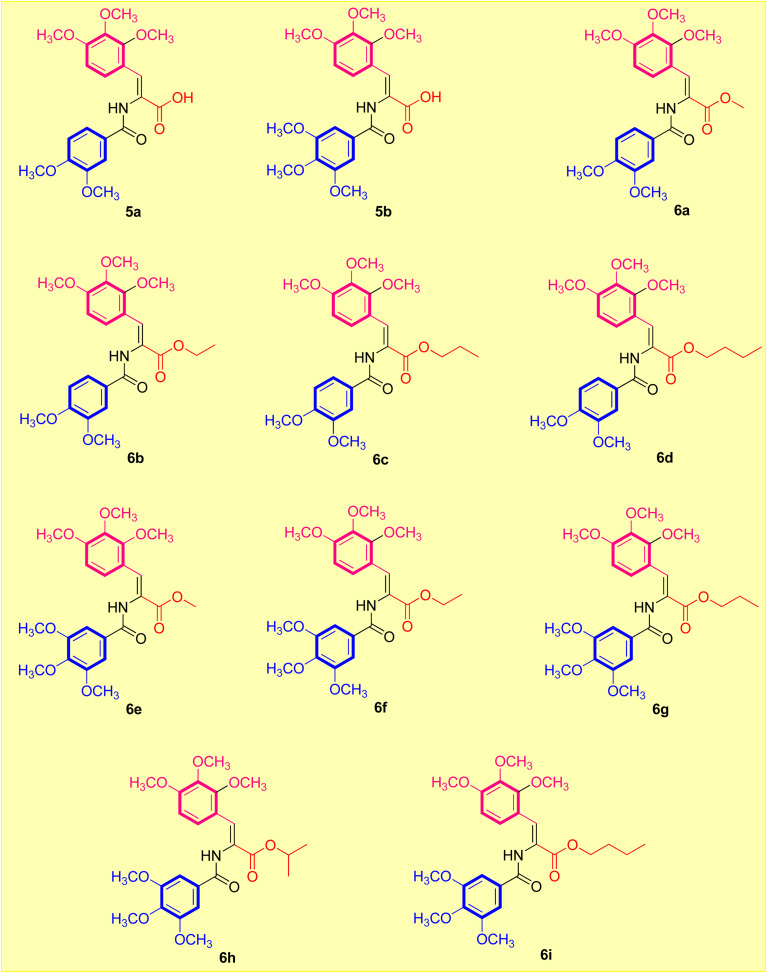
Designed acrylate compounds (5a,b and 6a–i) in the current study.

The two most frequently utilized methods in medicinal chemistry in the design of novel molecules are bioisosterism and molecular hybridization.^27^ The isosteric modification method is an efficient and often used technique that many drug candidates employ to enhance their pharmacodynamic behavior.^28^ It was of interest to further exploit the lead antimitotic agent, CA-4, with the possibility of producing effective drugs active against breast carcinoma cells.^29^ Encouraged by the above findings, we desired to design and synthesize a new series of acrylic acids and acrylate esters-containing scaffolds in the hope of getting more potent congeners (Fig. 1). The synthesized molecules were evaluated *in vitro* against MCF-7 breast cancer cells to assess their cytotoxic and β-tubulin polymerization inhibition activities (Fig. 2).

## 2. Results and discussion

### 2.1. Chemistry

**Scheme 1:**
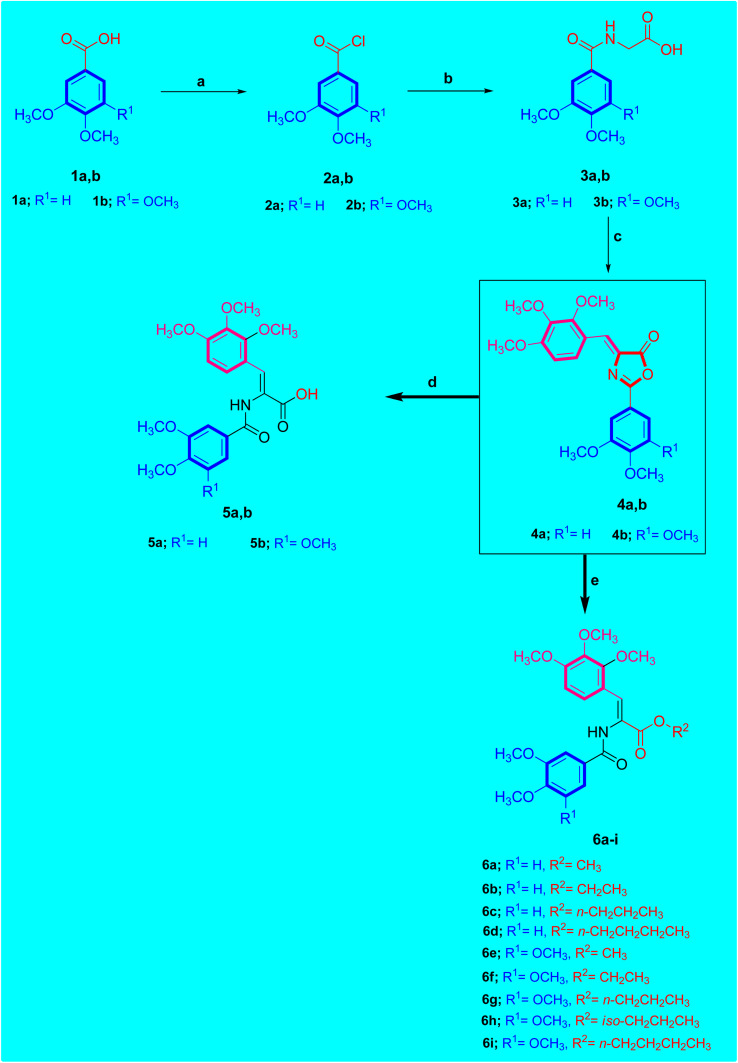
Synthetic approach of acrylate compounds 5a,b and 6a–i. Reagents: (a) SOCl_2_, CH_2_Cl_2_, reflux 2 h; (b) glycine, Et_3_N, CH_3_CN/H_2_O, stirring, r.t.; (c) 2,3,4-*tri*-OCH_3_-C_6_H_2_CHO, NaOAc, Ac_2_O, 80 °C 2 h; (d) KOH, H_2_O, reflux 3–4 h; (e) respective aliphatic alcohol, Et_3_N, reflux 2–3 h.

The targeted novel derivatives 5a,b and 6a–i were synthesized as depicted in Scheme 1. To prepare the key intermediates 4a,b, the Knoevenagel reaction was utilized. 2,3,4-Trimethoxybenzaldehyde was condensed with Respective hippuric acid 3a,b by refluxing in a solution of sodium acetate and acetic anhydride to give the desired (*Z*)-2-aryl-4-(2,3,4-trimethoxybenzylidene)oxazol-5(4*H*)-ones 4a,b.

Consequently, treatment of compound 4a,b with aqueous K_2_CO_3_ for 3–4 h followed by neutralization in acidic solution afforded the corresponding carboxylic acid derivative 5a,b. The ^1^H-NMR spectrum of acrylic acid compound 5b showed two singlet signals at *δ* 12.65 and 9.75 ppm corresponding to OH proton of carboxylic acid moiety and NH proton of amide function, respectively. The aromatic protons of the 2,3,4-trimethoxybenzylidene ring of compound 5b resonated as two doublet signals at *δ* 7.44 and 6.85 ppm integrating for one proton for each signal, respectively. In addition, the aromatic protons of 3,4,5-trimethoxyphenyl ring of compound 5b resonated as singlet signal at *δ* 7.32 ppm integrating for two protons. The olefinic proton resonated as singlet peak at *δ* 7.57 ppm integrating for one proton. Furthermore, ^13^C-NMR spectrum of acrylic acid compound 5b showed five peaks at *δ* 61.92, 60.90, 60.57, 56.52 and 56.42 ppm corresponding to six methoxy groups of 2,3,4-trimethoxybenzylidene in addition to 3,4,5-trimethoxyphenyl moieties and two peaks at *δ* 162.66 and 163.77 ppm related to two carbonyl groups (C=O) of carboxylic acid and amide moieties, respectively.

Treating compound 4a,b with respective alcohol; namely methyl alcohol, ethyl alcohol, propyl alcohol, iso-propyl alcohol or *n*-butyl alcohol in the presence of triethyl amine (Et_3_N) yielded the desired ester derivative 6a–i. Concerning ester derivatives 6a–i, single peak corresponding to amide (NH) group was found in the ^1^H-NMR spectrum between *δ* 9.91 and 9.79 ppm. For example, consider compound 6g which was designated as (*Z*)-propyl 2-(3,4,5-trimethoxybenzamido)-3-(2,3,4-trimethoxyphenyl)acrylate C_25_H_33_NO_9_ showed the presence of two singlet peaks at 9.88 and 7.53 ppm corresponding to amidic NH and olefinic protons, respectively. In this respect, the ^1^H-NMR spectrum of compound 6g revealed three sets of protons resonated as triplet at *δ* 4.11, sextet at *δ* 1.65 and as triplet at *δ* 0.91 ppm integrating for 2H, 2H, and 3H, respectively which was attributed to propyl function (OCH_2_CH_2_CH_3_). ^13^C-NMR spectrum of compound 6g confirmed the carbon skeleton due to the presence of three signals at *δ* 66.64, 22.08 and 10.75 ppm corresponding to propyl group (OCH_2_CH_2_CH_3_), in addition to five peaks at *δ* 61.94, 60.94, 60.56, 56.52 and 56.44 ppm corresponding to six methoxy (OCH_3_) functions. Furthermore, ^13^C-NMR spectrum of compound 6g revealed the presence of two singlet peaks at *δ* 165.91 and 165.58 ppm due to carbonyl groups (C=O) of ester and amide moieties respectively.

### 2.2. Biology

#### 2.2.1. Cytotoxic activity against MCF-7 breast cancer cell line

Cytotoxic activity of the synthesized acrylate derivatives 5a–6i was evaluated against the MCF-7 breast carcinoma cell line using the MTT colorimetric assay. CA-4 was included as a positive control. The results were summarized as IC_50_ values in Table 1. From the presented results, it was found that the tested acrylate derivatives 5a–6i showed considerable cytotoxic activity against MCF-7 cells with IC_50_ values 2.57–42.08 μM. Compounds 5b (IC_50_ = 5.12 μM), 6a (IC_50_ = 6.74 μM), 6e (IC_50_ = 2.57 μM), 6f (IC_50_ = 3.26 μM) and 6h (IC_50_ = 7.08 μM) were the most active molecules against MCF-7 cells. From the obtained results, compound 6e (IC_50_ = 2.57 μM) emerged as the most active member of the synthesized acrylate derivatives. In the acrylic acid analogs 5a,b, compound 5b (IC_50_ = 5.12 μM) bearing 3,4,5-trimethoxyphenyl and 2,3,4-trimethoxybenzylidene moieties displayed better cytotoxic activity than compound 5a (IC_50_ = 9.31 μM) bearing 3,4-dimethoxyphenyl and 2,3,4-trimethoxybenzylidene groups. Regarding the acrylate ester derivatives 6a–i, it can be observed that the methyl acrylate ester compound 6e (IC_50_ = 2.57 μM) exerted good cytotoxic activity and was the most active compound compared with its methyl acrylate ester congeners 6a (IC_50_ = 6.74 μM) or other alkyl substituted derivatives.

**Table 1:** *In vitro* cytotoxic activity of the synthesized acrylic acids 5a,b and acrylate esters 6a–i against MCF-7 breast carcinoma cell line^a^

Comp. no.	IC_50_ value (μM)	
	MCF-7	MCF-10A
5a	9.31 ± 0.35	NT
5b	5.12 ± 0.32	NT
6a	6.74 ± 0.78	NT
6b	17.08 ± 0.49	NT
6c	20.26 ± 0.44	NT
6d	42.08 ± 0.96	NT
6e	2.57 ± 0.16	19.06 ± 0.31
6f	3.26 ± 0.21	NT
6g	11.34 ± 0.75	NT
6h	7.08 ± 0.29	NT
6i	33.02 ± 1.03	NT
CA-4	1.25 ± 0.08	13.18 ± 0.19

a NT; not tested.

#### 2.2.2. Tubulin assay

**Fig. 3:**
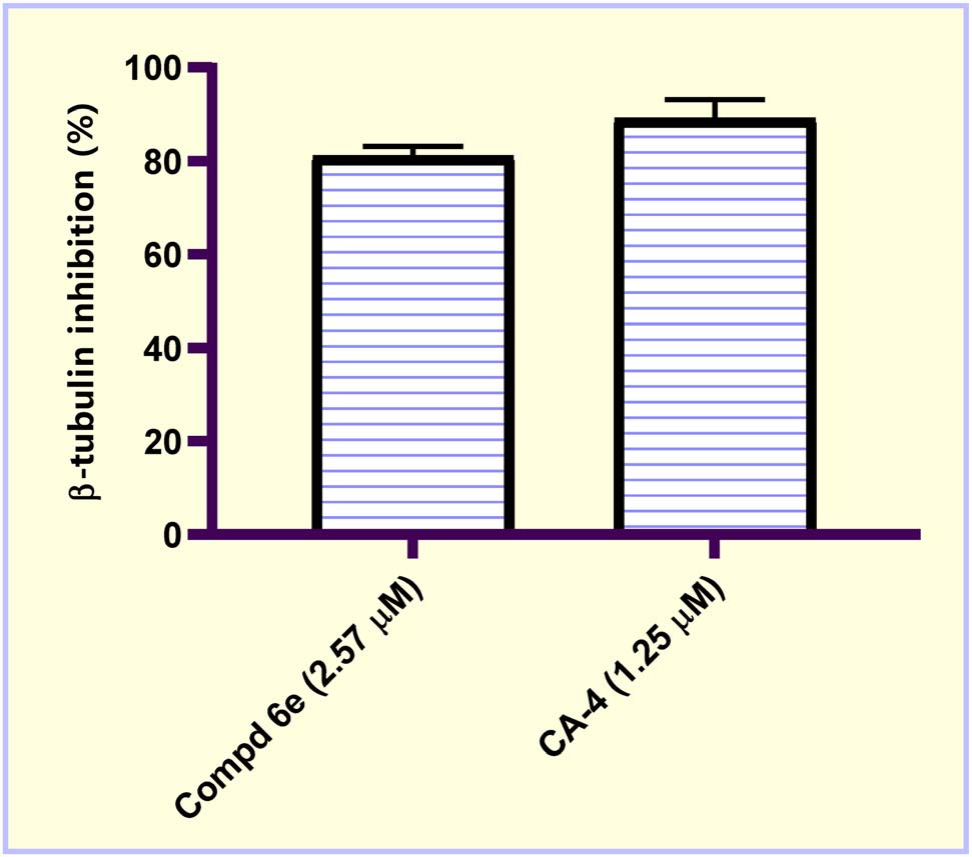
Graphical representation of β-tubulin inhibition (%) in MCF-7 breast carcinoma cells after treatment with methyl acrylate ester 6e and CA-4 at the IC_50_ concentration (μM).

The disruption of the cellular microtubule structure and, more importantly, microtubule function that result from inhibiting β-tubulin polymerization leads to the triggering of cellular apoptosis.^30,31^ Inhibition of tubulin polymerization has been identified as a possible therapeutic target for the creation of new cancer therapies.^32^ The most potent cytotoxic acrylate ester molecule 6e was assessed for its β-tubulin polymerization inhibition activity using CA-4 as a reference compound. This compound was tested at a concentration equal to its IC_50_ dose value (IC_50_ = 2.57 μM) for 48 h utilizing MCF-7 breast carcinoma cells. The results were presented as percent inhibition values, as shown in Fig. 3. The results showed that, as compared to untreated control cells, the tested acrylate ester compound 6e produced a 5.73-fold decrease in the level of β-tubulin polymerization. In addition, compared to CA-4 (89.17% polymerization inhibition), acrylate ester molecule 6e showed good β-tubulin polymerization inhibition activity with β-tubulin inhibition percentage of 81.16%. These results showed that the molecular target of acrylate ester molecule 6e may be tubulin.

#### 2.2.3. Cell cycle analysis

**Fig. 4:**
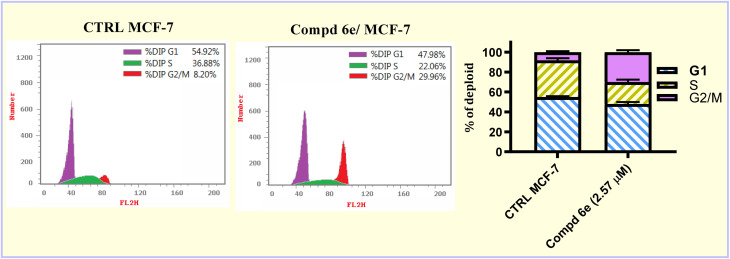
Flow cytometric cell cycle analysis of MCF-7 breast carcinoma cells before and after 48 h treatment with 2.57 μM of methyl acrylate ester 6e.

Tubulin polymerization inhibitors have demonstrated effectiveness in disrupting cellular cycle stages, resulting in cellular cycle arrest and cell death.^33^ To demonstrate the checkpoint at which the synthesized acrylate compounds block cellular growth, a cell cycle assay was carried out for the most active ester candidate, 6e. This compound was tested at a concentration equal to its IC_50_ dose value (IC_50_ = 2.57 μM) for 48 h utilizing MCF-7 breast carcinoma cells. It can be shown that methyl acrylate compound 6e revealed a significant decrease in the cellular population in the G1 and S phases from 54.92 and 26.88%, respectively (in control untreated cells) to 47.98 and 22.06%, respectively (in treated cells). On the other hand, the cell population increased in the G2/M phase from 8.20% (in control untreated cells) to 29.96% (in the treated cells) (Fig. 4). These findings indicated that methyl acrylate compound 6e arrested the MCF-7 breast carcinoma cells at the G2/M phase checkpoint.

#### 2.2.4. Apoptosis analysis

**Fig. 5:**
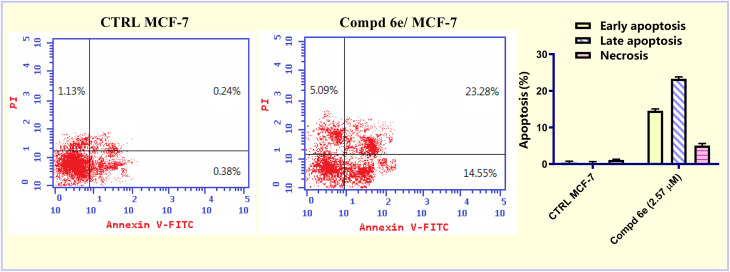
The impact of methyl acrylate ester 6e on the percentage of Annexin V-FITC positive staining in breast carcinoma MCF-7 cells before and after 48 h treatment with 2.57 μM.

Apoptosis is vital for maintaining proper tissue homeostasis within mature organisms.^34^ Apoptosis inhibition can contribute to the onset and spread of cancer, so apoptosis activation in cancer cells may have positive benefits in cancer chemotherapy.^35^ Fluorochrome Annexin-V/propidium iodide (PI) staining (Annexin-V/FITC) was performed to assess the apoptotic activity of the most active acrylate ester derivative 6e, utilizing MCF-7 breast carcinoma cells at a concentration of 2.57 μM for 48 h. The results in Fig. 5 demonstrated that, methyl acrylate ester 6e showed a significant increase in apoptotic activity. For the early stage, the apoptotic cells increased from 0.38% (in the control untreated cells) to 14.55% (in the treated cells). Regarding the late stage, the apoptotic cells increased from 0.24% (in the control untreated cells) to 23.28% (in the treated cells). These results revealed that acrylate ester 6e produced a 49.27-fold increase in the apoptotic cells compared to the control untreated MCF-7 breast carcinoma cells.

#### 2.2.5. *In vitr*o gene expression measurement for p53, Bax and Bcl-2

**Fig. 6:**
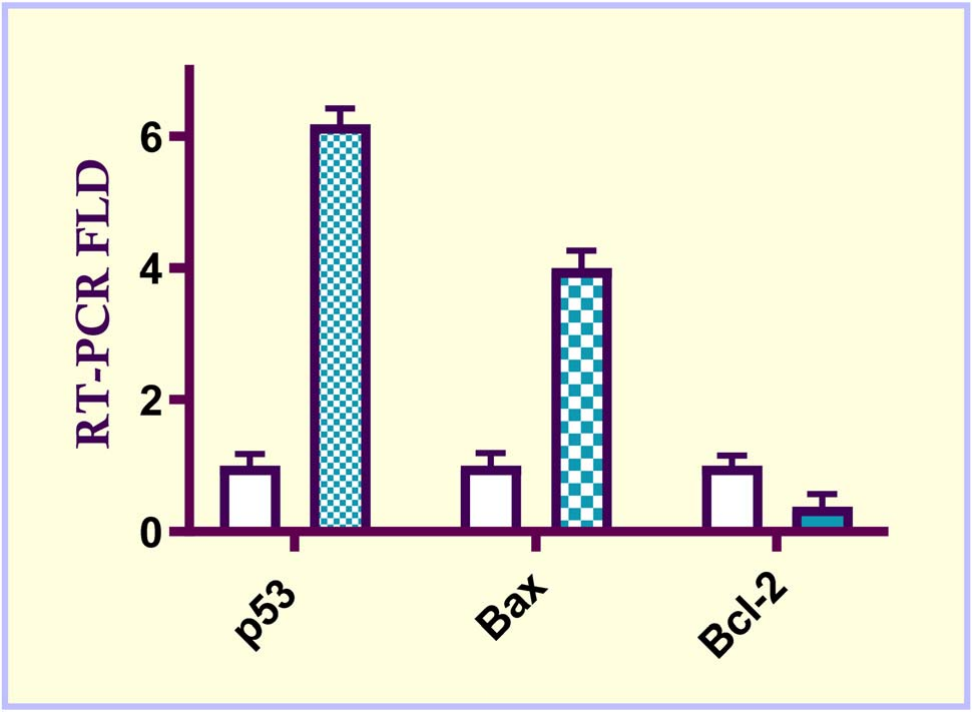
Effect of methyl acrylate ester 6e (IC_50_ = 2.57 μM) on the gene expression of apoptosis-associated markers before and after a 48 h treatment in MCF-7 breast carcinoma cells.

p53, Bax and Bcl-2 play a key role in controlling intrinsic mitochondrial cellular death.^36^ Molecular control of the apoptotic process may ultimately lead to the development of more potent therapies for cancer.^37^ During the apoptotic process, these mediators have opposing effects. p53 and Bax have proapoptotic impacts, while Bcl-2 has anti-apoptotic effects. At a dose value of 2.57 μM, the most active ester derivative 6e was tested for its intrinsic apoptosis in MCF-7 breast carcinoma cells. This assay utilized the quantitative real time reverse transcriptase PCR (qRT-PCR) assay. The data indicated that methyl acrylate ester derivatives 6e upregulate p53 level by 6.18-fold higher than untreated control cells. Similarly, acrylate ester compound 6e increased Bax level by 3.99-fold relative to untreated control cells. On the other hand, compound 6e decreased the level of anti-apoptotic gene Bcl-2 level by 0.38-fold less than untreated cells. Subsequently, compound 6e increased the ratio of Bax/Bcl-2 by 10.5-fold as compared to untreated cells. These findings demonstrated that the methyl acrylate ester derivative 6e has a strong apoptotic impact on the intrinsic mitochondrial apoptotic pathway (Fig. 6).

## 3. Conclusions

In the current study, a new series of acrylic acid and acrylate ester derivatives structurally related to CA-4 were designed and synthesized. The chemical structures of the constructed acrylate derivatives were substantiated on the basis of ^1^H-NMR, ^13^C-NMR spectroscopic studies and elemental analyses. The results revealed that methyl acrylate compound 6e (IC_50_ = 2.57 ± 0.16 μM) was the most potent against the MCF-7 breast carcinoma cell line. Methyl acrylate molecule 6e showed good β-tubulin polymerization inhibition activity (81.16% polymerization inhibition) relative to CA-4 as a positive control (82.82% polymerization inhibition). Compound 6e exerted an increase in the percentage of MCF-7 cells at the G2/M phase from 8.20% to 29.96% compared to the untreated control. In addition, methyl acrylate molecule 6e elicited a significant increase in the apoptotic power of MCF-7 cells. In the early stage from 0.38% to 14.55% and in the late stage from 0.24% to 23.28% compared to the untreated control. Moreover, compound 6e boosted the gene expression levels of both p53, Bax by 6.18- and 3.99-fold, respectively, relative to the untreated control. On the other hand, it caused a significant reduction in the Bcl-2 gene expression level by 0.38-fold relative to untreated MCF-7 breast carcinoma cells.

## 4. Experimental

### 4.1. Synthesis

#### 4.1.1. General method of synthesis of (*Z*)-2-aryl-3-(2,3,4-trimethoxyphenyl)acrylic acids (5a,b)

Respective oxazolone derivatives 4a,b (0.0015 mol) was heated with KOH in distilled water (30 mL) for 3–4 h, till formation of the corresponding potassium salt. The reaction was filtered while hot and the filtrate thus obtained was neutralized with HCl (2 N). The obtained precipitate was filtered, dried and crystallized from aqueous ethanol (70%) to afford acrylic acid 5a,b.

##### 4.1.1.1 (*Z*)-2-(3,4-Dimethoxybenzamido)-3-(2,3,4-trimethoxyphenyl)acrylic acid (5a)

White powder (0.30 g, 48%), mp 201–203 °C. ^1^H-NMR (400 MHz, DMSO-*d*_6_, *δ* ppm): 12.60 (s, 1H, OH), 9.65 (s, 1H, NH), 7.65–7.59 (m, 1H, arom. CH), 7.55 (s, 2H, arom. CH and olefinic CH), 7.44 (d, *J* = 8.9 Hz, 1H, arom. CH), 7.07 (d, *J* = 8.5 Hz, 1H, arom. CH), 6.83 (d, *J* = 9.0 Hz, 1H, arom. CH), 3.85 (s, 3H, OCH_3_), 3.84 (s, 3H, OCH_3_), 3.82 (s, 3H, OCH_3_), 3.79 (s, 3H, OCH_3_), 3.76 (s, 3H, OCH_3_). ^13^C-NMR (100 MHz, DMSO-*d*_6_, *δ* ppm): 167.07, 165.80, 154.93, 152.79, 152.15, 148.77, 142.00, 127.26, 126.82, 126.29, 124.44, 121.55, 120.64, 111.43, 111.42, 108.57, 61.92, 60.91, 56.40, 56.14, 56.04. Anal. calcd for C_21_H_23_NO_8_ (417.41): C, 60.43; H, 5.55; N, 3.36. Found: C, 60.32; H, 5.42; N, 3.44.

##### 4.1.1.2 (*Z*)-2-(3,4,5-Trimethoxybenzamido)-3-(2,3,4-trimethoxyphenyl)acrylic acid (5b)

White powder (0.29 g, 43%), mp 217–219 °C. ^1^H-NMR (400 MHz, DMSO-*d*_6_, *δ* ppm): 12.65 (s, 1H, OH), 9.75 (s, 1H, NH), 7.57 (s, 1H, olefinic CH), 7.44 (d, *J* = 8.9 Hz, 1H, arom. CH), 7.32 (s, 2H, arom. CH), 6.85 (d, *J* = 9.0 Hz, 1H, arom. CH), 3.86 (s, 3H, OCH_3_), 3.85 (s, 6H, 2OCH_3_), 3.79 (s, 3H, OCH_3_), 3.76 (s, 3H, OCH_3_), 3.73 (s, 3H, OCH_3_). ^13^C-NMR (100 MHz, DMSO-*d*_6_, *δ* ppm): 166.95, 165.64, 155.00, 153.12, 152.81, 142.01, 140.86, 129.10, 127.36, 126.60, 124.45, 120.53, 108.63, 105.73, 61.92, 60.90, 60.57, 56.52, 56.42. Anal. calcd for C_22_H_25_NO_9_ (447.44): C, 59.06; H, 5.63; N, 3.13. Found: C, 58.85; H, 5.87; N, 3.19.

#### 4.1.2. General method of synthesis of (*Z*)-2-aryl-3-(2,3,4-trimethoxyphenyl)acrylate esters (6a–i)

A mixture of respective oxazolone derivatives 4a,b (0.0015 mol) and 25 mL of suitable aliphatic alcohol; namely methyl alcohol, ethyl alcohol, *n*-propyl alcohol, isopropyl alcohol and *n*-butyl alcohol contain triethyl amine (10 drops) was refluxed for 2–3 h. After reaction accomplishment, the reaction mixture was cooled and poured on crushed ice. The formed precipitate was filtered, dried and crystallized from ethanol (70%) to afford acrylate ester compounds 6a–i.

##### 4.1.2.1 (*Z*)-Methyl 2-(3,4-dimethoxybenzamido)-3-(2,3,4-trimethoxyphenyl)acrylate (6a)

White powder (0.45 g, 69%), mp 177–179 °C. ^1^H-NMR (400 MHz, DMSO-*d*_6_, *δ* ppm): 9.81 (s, 1H, NH), 7.63 (d, *J* = 8.4 Hz, 1H, arom. CH), 7.55 (s, 1H, olefinic CH), 7.51 (s, 1H, arom. CH), 7.44 (d, *J* = 8.9 Hz, 1H, arom. CH), 7.08 (d, *J* = 8.4 Hz, 1H, arom. CH), 6.85 (d, *J* = 9.0 Hz, 1H), 3.86 (s, 3H, OCH_3_), 3.84 (s, 3H, OCH_3_), 3.83 (s, 3H, OCH_3_), 3.80 (s, 3H, OCH_3_), 3.76 (s, 3H, OCH_3_), 3.73 (s, 3H, OCH_3_). ^13^C-NMR (100 MHz, DMSO-*d*_6_, *δ* ppm): 166.22, 165.91, 155.15, 152.88, 152.29, 148.83, 142.02, 127.37, 126.06, 125.95, 124.53, 121.61, 120.31, 111.48, 111.41, 108.62, 61.96, 60.91, 56.44, 56.16, 56.05, 52.65. Anal. calcd for C_22_H_25_NO_8_ (431.44): C, 61.25; H, 5.84; N, 3.25. Found: C, 61.33; H, 6.04; N, 3.13.

##### 4.1.2.2 (*Z*)-Ethyl 2-(3,4-dimethoxybenzamido)-3-(2,3,4-trimethoxyphenyl)acrylate (6b)

White powder (0.41 g, 61%), mp 154–156 °C. ^1^H-NMR (400 MHz, DMSO-*d*_6_, *δ* ppm): 9.78 (s, 1H, NH), 7.68–7.60 (m, 1H, arom. CH), 7.55 (s, 1H, olefinic CH), 7.50 (s, 1H, arom. CH), 7.45 (d, *J* = 8.5 Hz, 1H, arom. CH), 7.08 (d, *J* = 7.7 Hz, 1H, arom. CH), 6.85 (d, *J* = 8.4 Hz, 1H, arom. CH), 4.19 (q, *J* = 1.6 Hz, 2H, OCH_2_CH_3_), 3.86 (s, 3H, OCH_3_), 3.84 (s, 3H, OCH_3_), 3.83 (s, 3H, OCH_3_), 3.80 (s, 3H, OCH_3_), 3.76 (s, 3H, OCH_3_), 1.23 (t, *J* = 1.3 Hz, 3H, OCH_2_CH_3_). ^13^C-NMR (100 MHz, DMSO-*d*_6_, *δ* ppm): 165.95, 165.66, 155.08, 152.86, 152.25, 148.82, 142.02, 127.06, 126.38, 126.09, 124.53, 121.57, 120.39, 111.48, 111.39, 108.60, 61.95 (OCH_2_CH_3_), 61.19, 60.91, 56.42, 56.15, 56.05, 14.62 (OCH_2_CH_3_). Anal. calcd for C_23_H_27_NO_8_ (445.46): C, 62.01; H, 6.11; N, 3.14. Found: C, 61.88; H, 6.16; N, 3.02.

##### 4.1.2.3 (*Z*)-Propyl 2-(3,4-dimethoxybenzamido)-3-(2,3,4-trimethoxyphenyl)acrylate (6c)

White powder (0.44 g, 64%), mp 119–121 °C. ^1^H-NMR (400 MHz, DMSO-*d*_6_, *δ* ppm): 9.79 (s, 1H, NH), 7.62 (d, *J* = 7.0 Hz, 1H, arom. CH), 7.54 (s, 1H, olefinic CH), 7.51 (s, 1H, arom. CH), 7.46 (d, *J* = 8.9 Hz, 1H, arom. CH), 7.08 (d, *J* = 8.5 Hz, 1H, arom. CH), 6.85 (d, *J* = 9.0 Hz, 1H, arom. CH), 4.10 (t, *J* = 6.4 Hz, 2H, OCH_2_CH_2_CH_3_), 3.85 (s, 3H, OCH_3_), 3.84 (s, 3H, OCH_3_), 3.82 (s, 3H, OCH_3_), 3.80 (s, 3H, OCH_3_), 3.76 (s, 3H, OCH_3_), 1.62 (h, *J* = 7.0 Hz, 2H, OCH_2_CH_2_CH_3_), 0.90 (t, *J* = 7.4 Hz, 3H, OCH_2_CH_2_CH_3_). ^13^C-NMR (100 MHz, DMSO-*d*_6_, *δ* ppm): 166.03, 165.73, 155.10, 152.87, 152.23, 148.80, 142.04, 127.09, 126.37, 126.12, 124.55, 121.56, 120.38, 111.48, 111.38, 108.64, 66.59 (OCH_2_CH_2_CH_3_), 61.94, 60.92, 56.43, 56.15, 56.05, 22.08 (OCH_2_CH_2_CH_3_), 10.76 (OCH_2_CH_2_CH_3_). Anal. calcd for C_24_H_29_NO_8_ (459.49): C, 62.73; H, 6.36; N, 3.05. Found: C, 62.86; H, 6.29; N, 2.93.

##### 4.1.2.4 (*Z*)-Butyl 2-(3,4-dimethoxybenzamido)-3-(2,3,4-trimethoxyphenyl)acrylate (6d)

White powder (0.37 g, 52%), mp 132–134 °C. ^1^H-NMR (400 MHz, DMSO-*d*_6_, *δ* ppm): 9.81 (s, 1H, NH), 7.66–7.60 (m, 1H, arom. CH), 7.57–7.53 (m, 1H, arom. CH), 7.51 (s, 1H, olefinic CH), 7.44 (d, *J* = 9.0 Hz, 1H, arom. CH), 7.08 (d, *J* = 8.5 Hz, 1H, arom. CH), 6.85 (d, *J* = 9.0 Hz, 1H, arom. CH), 4.31–3.91 (m, 2H, OCH_2_CH_2_CH_2_CH_3_), 3.86 (s, 3H, OCH_3_), 3.84 (s, 3H, OCH_3_), 3.83 (s, 3H, OCH_3_), 3.80 (s, 3H, OCH_3_), 3.76 (s, 3H, OCH_3_), 3.73 (s, 2H, OCH_2_CH_2_CH_2_CH_3_), 1.62 (q, *J* = 6.9 Hz, 2H, OCH_2_CH_2_CH_2_CH_3_), 0.90 (t, *J* = 7.4 Hz, 3H, OCH_2_CH_2_CH_2_CH_3_). ^13^C-NMR (100 MHz, DMSO-*d*_6_, *δ* ppm): 166.22, 165.91, 155.15, 152.88, 152.29, 148.83, 142.02, 127.36, 126.06, 125.96, 124.53, 121.61, 120.31, 111.48, 111.41, 108.62, 66.59 (OCH_2_CH_2_CH_2_CH_3_), 61.96, 60.91, 56.44, 56.16, 56.05, 52.65 (OCH_2_CH_2_CH_2_CH_3_), 22.09 (OCH_2_CH_2_CH_2_CH_3_), 10.76 (OCH_2_CH_2_CH_2_CH_3_). Anal. calcd for C_25_H_31_NO_8_ (473.52): C, 63.41; H, 6.60; N, 2.96. Found: C, 63.26; H, 6.68; N, 3.08.

##### 4.1.2.5 (*Z*)-Methyl 2-(3,4,5-trimethoxybenzamido)-3-(2,3,4-trimethoxyphenyl)acrylate (6e)

White powder (0.50 g, 72%), mp 133–135 °C. ^1^H-NMR (400 MHz, DMSO-*d*_6_, *δ* ppm): 9.91 (s, 1H, NH), 7.53 (s, 1H, olefinic CH), 7.44 (d, *J* = 8.9 Hz, 1H, arom. CH), 7.33 (s, 2H, arom. CH), 6.87 (d, *J* = 9.0 Hz, 1H, arom. CH), 3.86 (s, 3H, OCH_3_), 3.85 (s, 6H, 2OCH_3_), 3.80 (s, 3H, OCH_3_), 3.76 (s, 3H, OCH_3_), 3.74 (s, 6H, 2OCH_3_). ^13^C-NMR (100 MHz, DMSO-*d*_6_, *δ* ppm): 166.10, 165.76, 155.23, 153.17, 152.91, 142.02, 140.99, 128.75, 127.48, 125.82, 124.55, 120.18, 108.68, 105.77, 61.97, 60.91, 60.57, 56.53, 56.45, 52.70. Anal. calcd for C_23_H_27_NO_9_ (461.46): C, 59.86; H, 5.90; N, 3.04. Found: C, 59.98; H, 5.81; N, 2.93.

##### 4.1.2.6 (*Z*)-Ethyl 2-(3,4,5-trimethoxybenzamido)-3-(2,3,4-trimethoxyphenyl)acrylate (6f)

White powder (0.51 g, 71%), mp 127–129 °C. ^1^H-NMR (400 MHz, DMSO-*d*_6_, *δ* ppm): 9.87 (s, 1H, NH), 7.50 (s, 1H, olefinic CH), 7.44 (d, *J* = 8.9 Hz, 1H, arom. CH), 7.31 (s, 2H, arom. CH), 6.86 (d, *J* = 9.0 Hz, 1H, arom. CH), 4.19 (q, *J* = 7.1 Hz, 2H, OCH_2_CH_3_), 3.85 (s, 3H, OCH_3_), 3.84 (s, 6H, 2OCH_3_), 3.79 (s, 3H, OCH_3_), 3.75 (s, 3H, OCH_3_), 3.73 (s, 3H, OCH_3_), 1.23 (t, *J* = 7.1 Hz, 3H, OCH_2_CH_3_). ^13^C-NMR (100 MHz, DMSO-*d*_6_, *δ* ppm): 165.80, 165.53, 155.17, 153.16, 152.88, 142.02, 140.95, 128.91, 127.22, 126.13, 124.54, 120.25, 108.67, 105.73, 61.95 (OCH_2_CH_3_), 61.25, 60.91, 60.56, 56.53, 56.44, 14.64 (OCH_2_CH_3_). Anal. calcd for C_24_H_29_NO_9_ (475.49): C, 60.62; H, 6.15; N, 2.95. Found: C, 60.53; H, 6.22; N, 3.03.

##### 4.1.2.7 (*Z*)-Propyl 2-(3,4,5-trimethoxybenzamido)-3-(2,3,4-trimethoxyphenyl)acrylate (6g)

White powder (0.46 g, 63%), mp 111–113 °C. ^1^H-NMR (400 MHz, DMSO-*d*_6_, *δ* ppm): 9.88 (s, 1H, NH), 7.53 (s, 1H, olefinic CH), 7.46 (d, *J* = 8.9 Hz, 1H, arom. CH), 7.31 (s, 2H, arom. CH), 6.88 (d, *J* = 9.0 Hz, 1H, arom. CH), 4.11 (t, *J* = 6.4 Hz, 2H, OCH_2_CH_2_CH_3_), 3.86 (s, 3H, OCH_3_), 3.85 (s, 6H, 2OCH_3_), 3.80 (s, 3H, OCH_3_), 3.76 (s, 3H, OCH_3_), 3.74 (s, 3H, OCH_3_), 1.63 (h, *J* = 7.2 Hz, 2H, OCH_2_CH_2_CH_3_), 0.91 (t, *J* = 7.4 Hz, 3H, OCH_2_CH_2_CH_3_). ^13^C-NMR (100 MHz, DMSO-*d*_6_, *δ* ppm): 165.91, 165.58, 155.18, 153.14, 152.89, 142.03, 140.91, 128.95, 127.26, 126.09, 124.56, 120.23, 108.69, 105.71, 66.64 (OCH_2_CH_2_CH_3_), 61.94, 60.94, 60.56, 56.52, 56.44, 22.08 (OCH_2_CH_2_CH_3_), 10.75 (OCH_2_CH_2_CH_3_). Anal. calcd for C_25_H_31_NO_9_ (489.51): C, 61.34; H, 6.38; N, 2.86. Found: C, 61.42; H, 6.29; N, 3.02.

##### 4.1.2.8 (*Z*)-Isopropyl 2-(3,4,5-trimethoxybenzamido)-3-(2,3,4-trimethoxyphenyl)acrylate (6h)

Pale yellow powder (0.44 g, 60%), mp 105–107 °C. ^1^H-NMR (400 MHz, DMSO-*d*_6_, *δ* ppm): 9.84 (s, 1H, NH), 7.47 (s, 1H, olefinic CH), 7.44 (d, *J* = 8.9 Hz, 1H, arom. CH), 7.30 (s, 2H, arom. CH), 6.87 (d, *J* = 9.0 Hz, 1H, arom. CH), 4.99 (dt, *J* = 12.5, 6.2 Hz, 1H, OCH(CH_3_)_2_), 3.85 (s, 3H, OCH_3_), 3.85 (s, 6H, 2OCH_3_), 3.80 (s, 3H, OCH_3_), 3.76 (s, 3H, OCH_3_), 3.74 (s, 3H, OCH_3_), 1.24 (d, *J* = 6.2 Hz, 6H, OCH(CH_3_)_2_). ^13^C-NMR (100 MHz, DMSO-*d*_6_, *δ* ppm): 165.83, 165.05, 155.10, 153.15, 152.85, 142.02, 140.90, 129.04, 126.86, 126.48, 124.54, 120.31, 108.66, 105.69, 68.70 (OCH(CH_3_)_2_), 61.94, 60.91, 60.56, 56.53, 56.43, 22.11 (OCH(CH_3_)_2_). Anal. calcd for C_25_H_31_NO_9_ (489.51): C, 61.34; H, 6.38; N, 2.86. Found: C, 61.39; H, 6.44; N, 2.77.

##### 4.1.2.9 Butyl 2-(3,4,5-trimethoxybenzamido)-3-(2,3,4-trimethoxyphenyl)acrylate (6i)

White powder (0.39 g, 52%), mp 118–120 °C. ^1^H-NMR (400 MHz, DMSO-*d*_6_, *δ* ppm): 9.90 (d, *J* = 11.2 Hz, 1H, NH), 7.53 (d, *J* = 4.4 Hz, 1H, arom. CH), 7.44 (d, *J* = 8.8 Hz, 1H, arom. CH), 7.32 (d, *J* = 6.7 Hz, 2H, arom. CH), 6.87 (dd, *J* = 9.0, 1.8 Hz, 1H, arom. CH), 4.22–3.96 (m, 2H, OCH_2_CH_2_CH_2_CH_3_), 3.86 (s, 3H, OCH_3_), 3.85 (s, 6H, 2OCH_3_), 3.80 (s, 3H, OCH_3_), 3.76 (s, 3H, OCH_3_), 3.74 (s, 3H, OCH_3_), 3.74 (s, 2H, OCH_2_CH_2_CH_2_CH_3_), 1.64 (dt, *J* = 13.9, 7.0 Hz, 2H, OCH_2_CH_2_CH_2_CH_3_), 0.91 (t, *J* = 7.4 Hz, 3H, OCH_2_CH_2_CH_2_CH_3_). ^13^C-NMR (100 MHz, DMSO-*d*_6_, *δ* ppm): 166.10, 165.77, 155.23, 153.15, 152.91, 142.04, 141.00, 128.75, 127.48, 125.83, 124.55, 120.18, 108.68, 105.77, 66.65 (OCH_2_CH_2_CH_2_CH_3_), 61.96, 60.91, 60.57, 56.53, 56.45, 52.69 (OCH_2_CH_2_CH_2_CH_3_), 22.09 (OCH_2_CH_2_CH_2_CH_3_), 10.75 (OCH_2_CH_2_CH_2_CH_3_). Anal. calcd for C_26_H_33_NO_9_ (503.54): C, 62.02; H, 6.61; N, 2.78. Found: C, 61.89; H, 6.67; N, 2.91.

### 4.2. Biological study

#### 4.2.1. Antiproliferative activity on MCF-7 cells

Antiproliferative activity of the constructed acrylate molecules 5a–6i was determined on MCF-7 cell line as reported previously.^38^ See ESI Appendix A.†

#### 4.2.2. Tubulin polymerization assay

The tubulin polymerization assay kit was used to measure the effect of the acrylate compounds 6e and CA-4 on tubulin polymerization. See ESI Appendix A.†

#### 4.2.3. Cell cycle analysis

The MCF-7 cell line was used for cell cycle analysis. Assay was carried out as reported previously.^39^ See ESI Appendix A.†

#### 4.2.4. Apoptosis assay

The MCF-7 cell line was used for Annexin V apoptosis assay. See ESI Appendix A.†

#### 4.2.5. Impact on the expression levels of apoptosis related markers

The MCF-7 cell line was used for measurement of the expression levels of Apoptotic markers. See Section S4.2.5 in ESI.†

## Conflicts of interest

The authors declare no conflict of interest.

## Supplementary Material

RA-013-D3RA03849A-s001
