# Supplementary material for: Design, synthesis and antiproliferative screening of newly synthesized acrylate derivatives as potential anticancer agents
Source: RSC Adv. 2023 Aug 4;13(34):23538–46. doi: 10.1039/d3ra03849a (PMC10402871; doi:10.1039/d3ra03849a)
Supplement: RA-013-D3RA03849A-s001 [file RA-013-D3RA03849A-s001.pdf]

Electronic Supplementary Material (ESI) for RSC Advances.
This journal is © The Royal Society of Chemistry 2023

Design, Synthesis and Antiproliferative Screening of Newly Synthesized Acrylate Derivatives as Potential Anticancer Agents

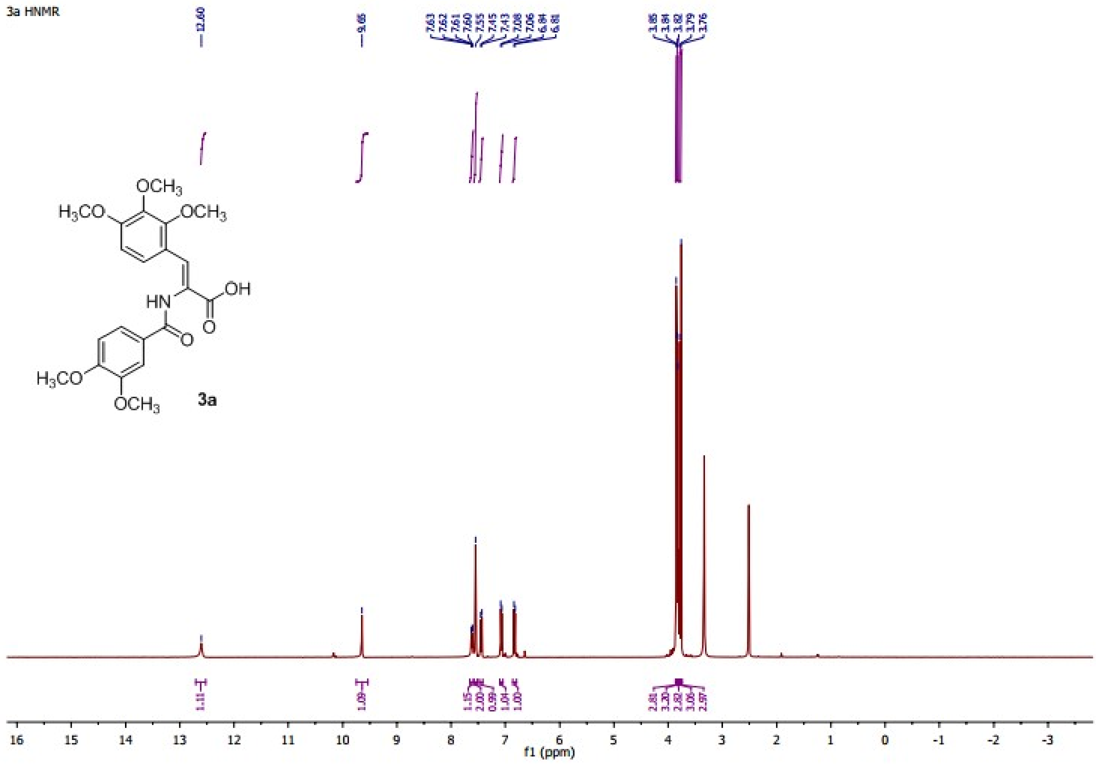

Figure S1: ^{1}H-NMR spectrum of compound **5a**

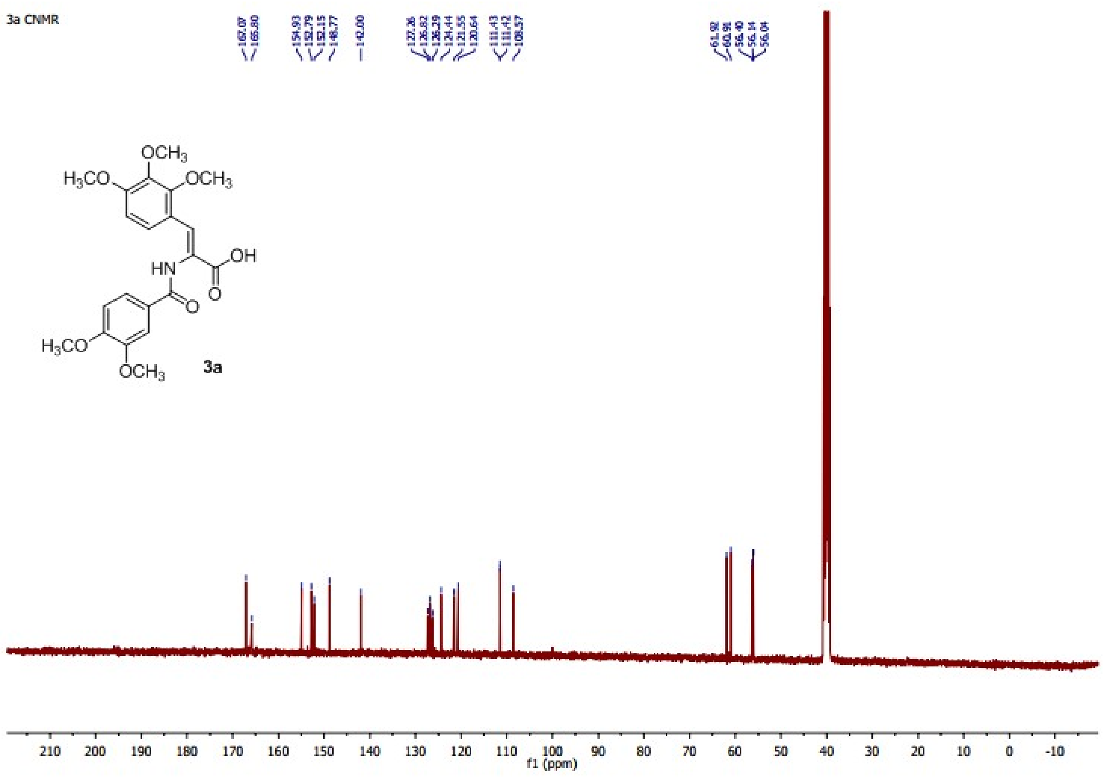

Figure S2: ^{13}C-NMR spectrum of compound **5a**

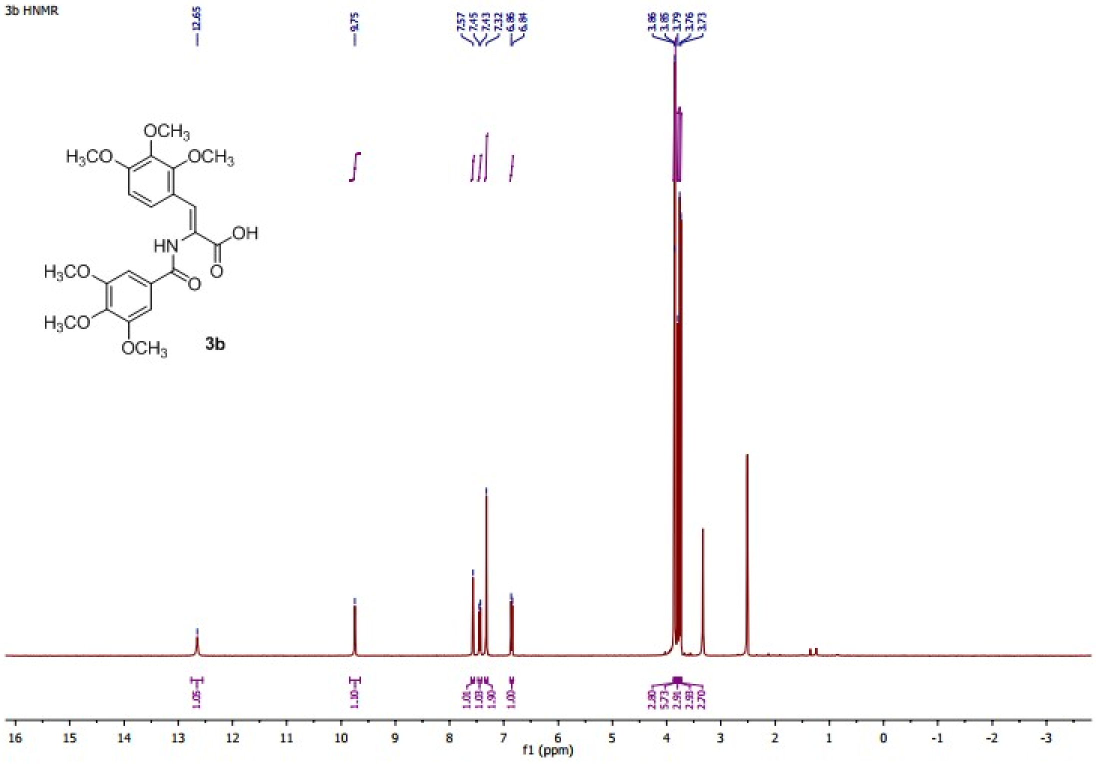

Figure S3: ^{1}H-NMR spectrum of compound **5b**

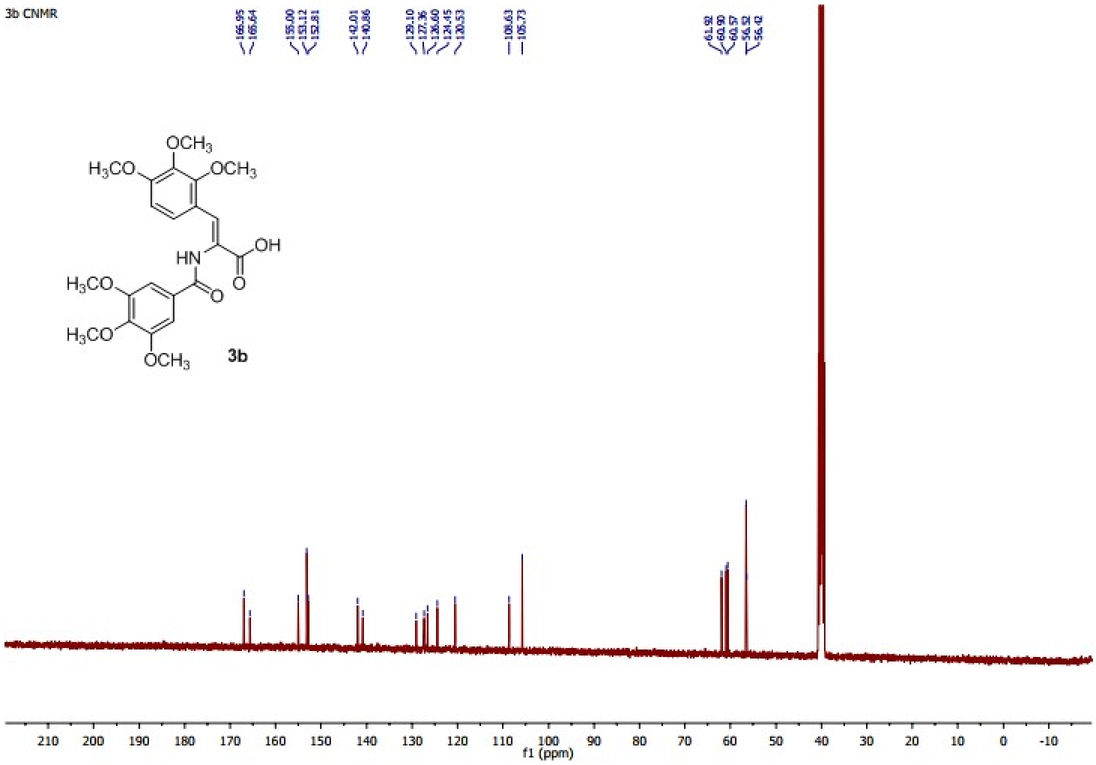

Figure S4: ^{13}C-NMR spectrum of compound **5b**

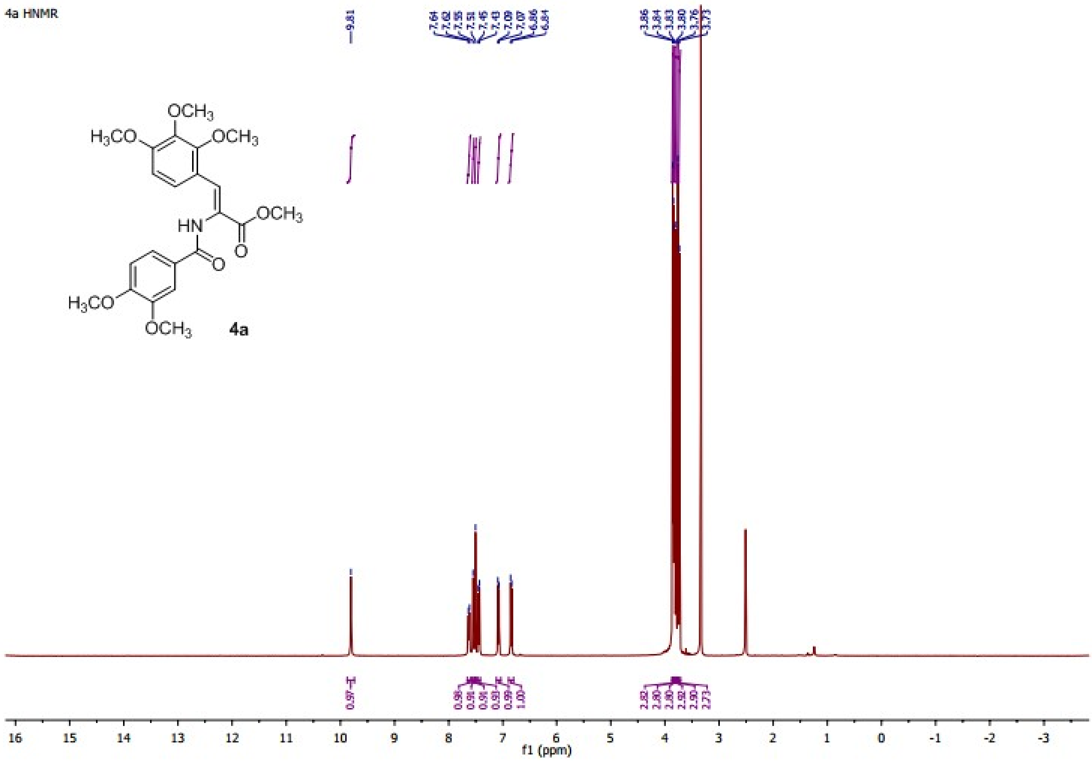

Figure S5: ^{1}H-NMR spectrum of compound **6a**

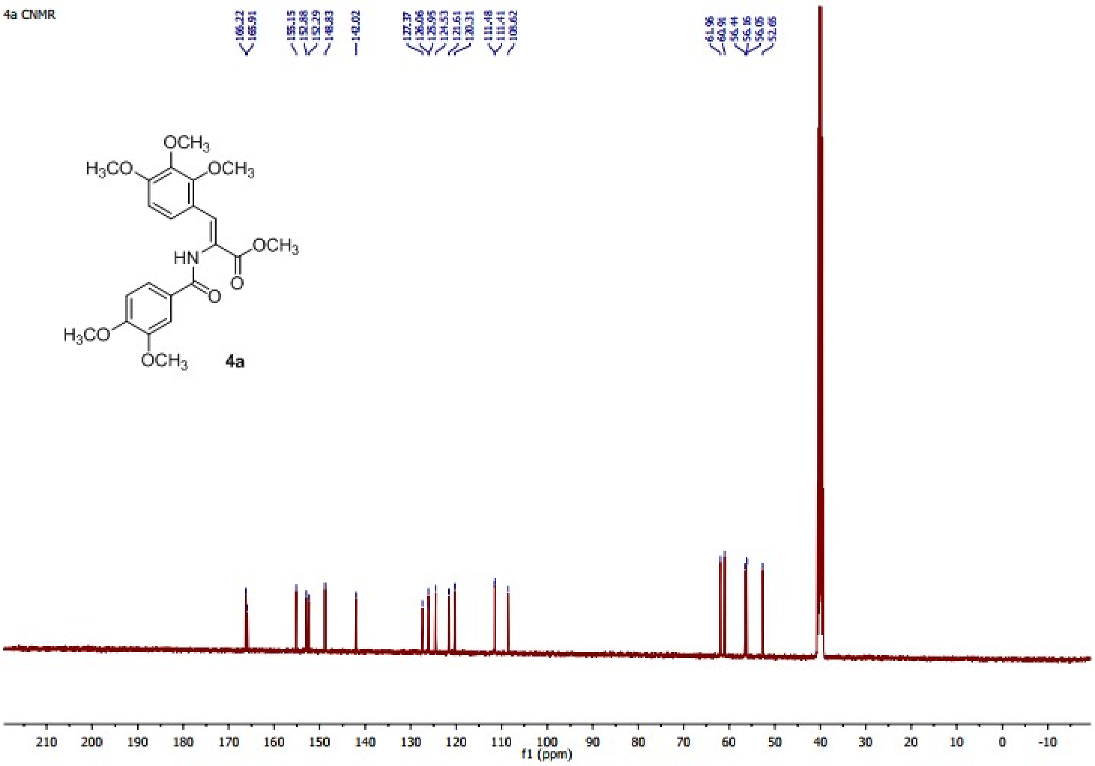

Figure S6: ^{13}C-NMR spectrum of compound **6a**

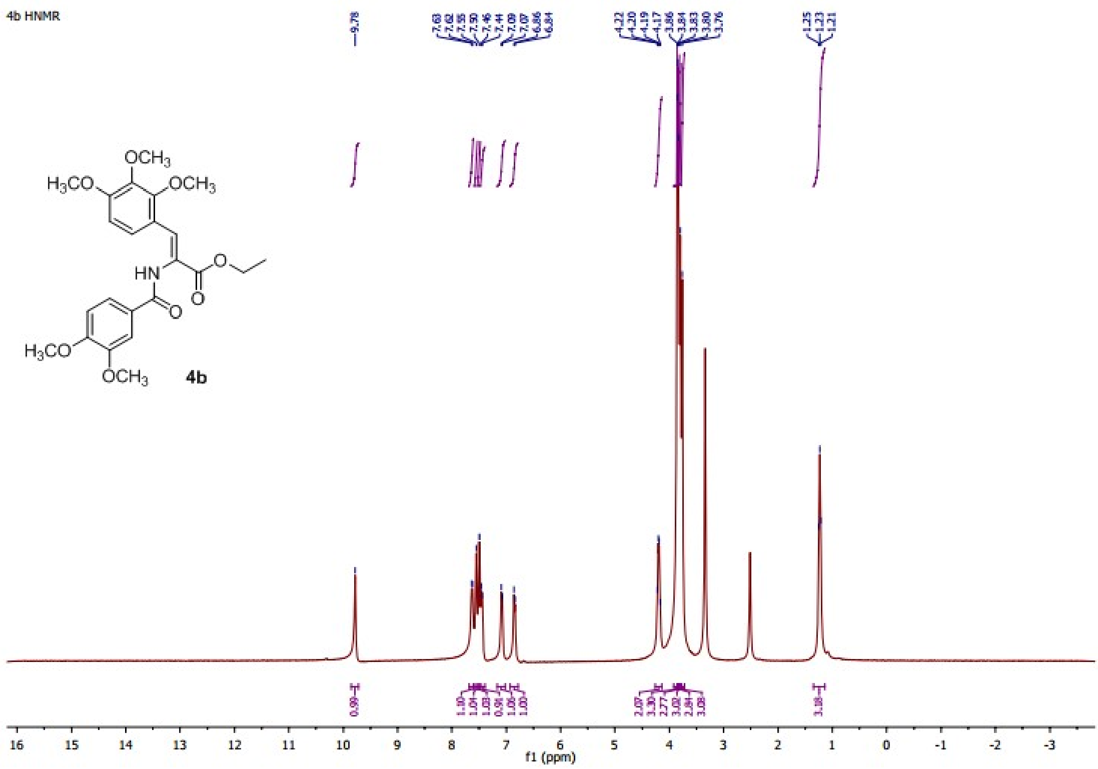

Figure S7: ^{1}H-NMR spectrum of compound **6b**

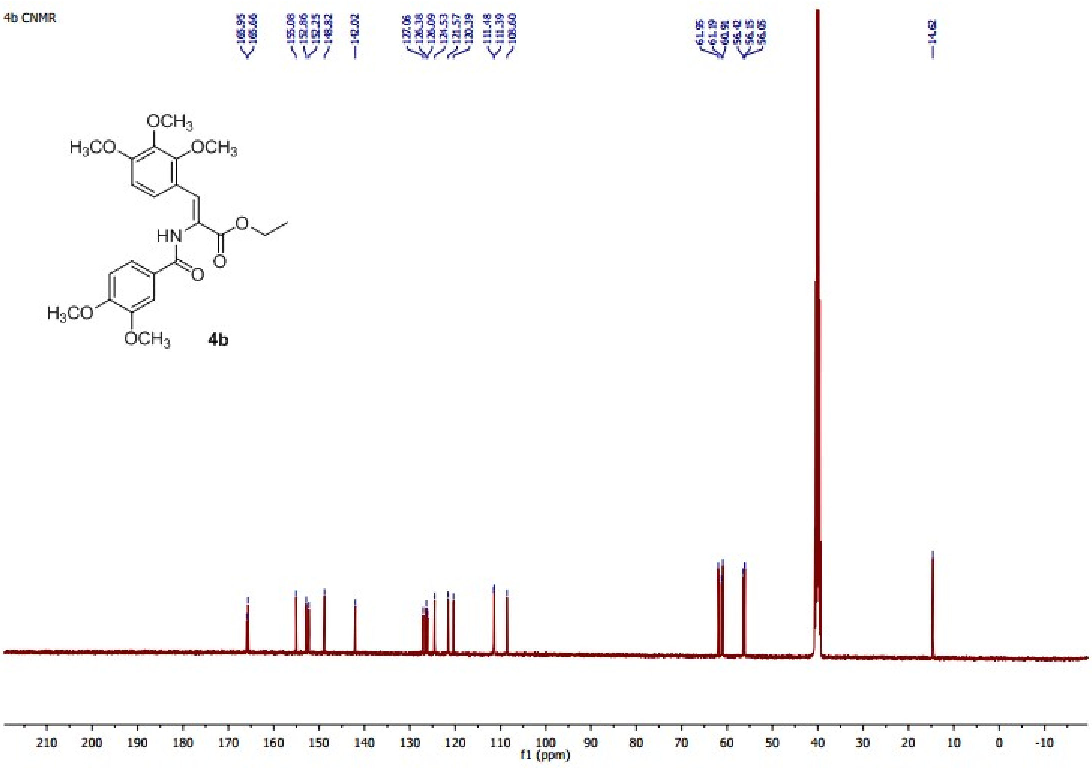

Figure S8: ^{13}C-NMR spectrum of compound **6b**

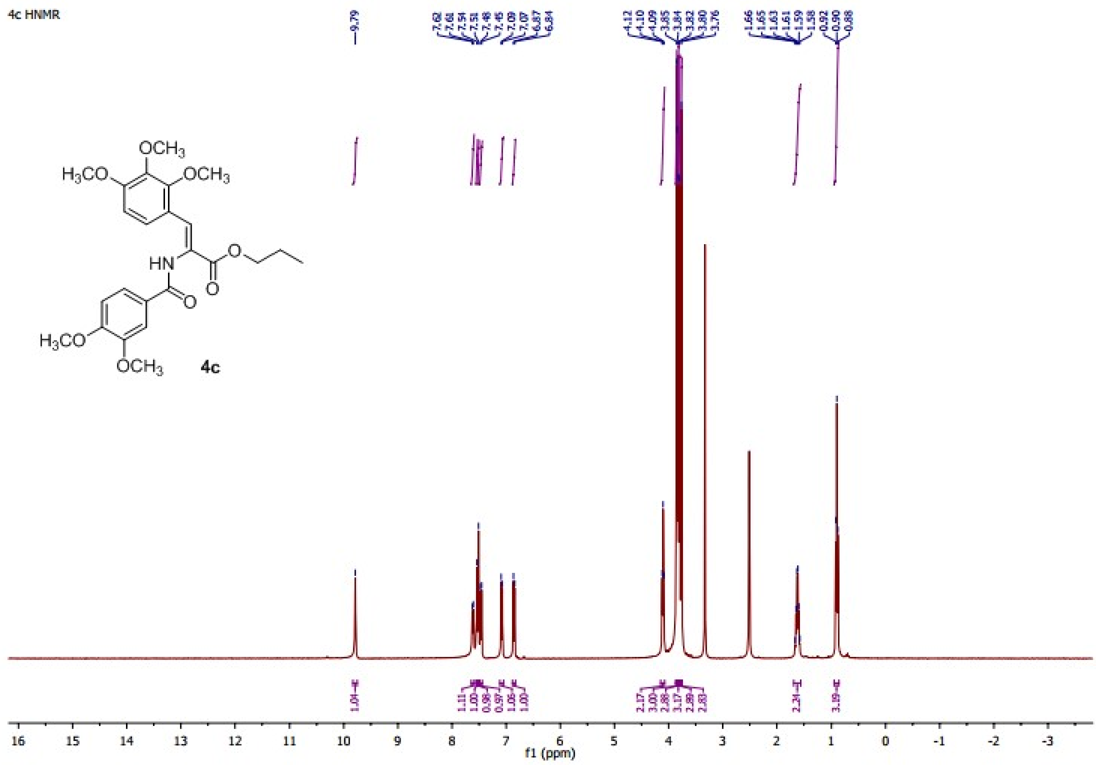

Figure S9: ^{1}H-NMR spectrum of compound **6c**

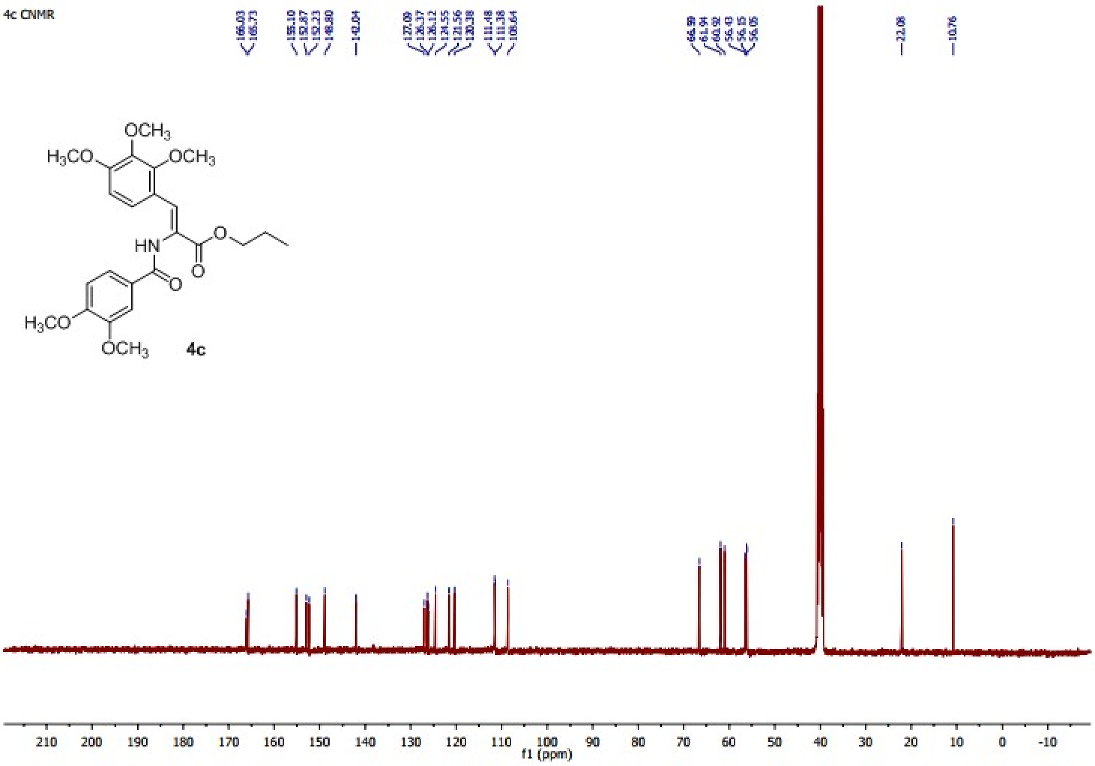

Figure S10: ^{13}C-NMR spectrum of compound **6c**

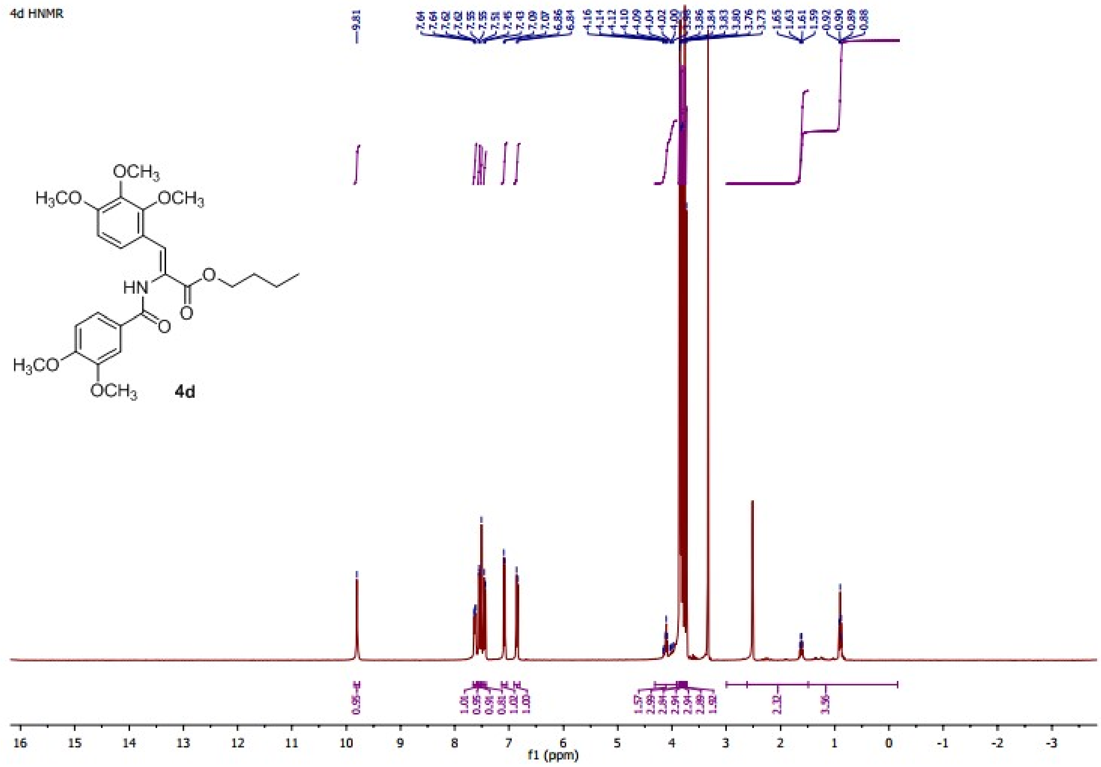

Figure S11: ^{1}H-NMR spectrum of compound **6d**

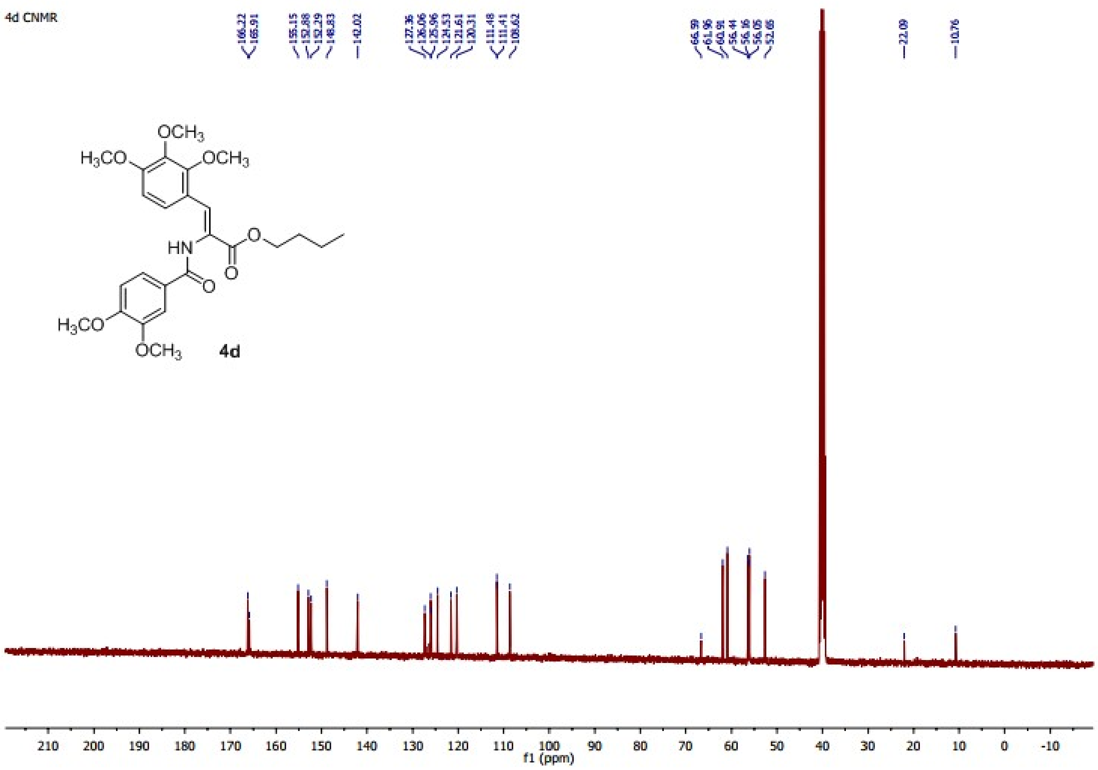

Figure S12: ^{13}C-NMR spectrum of compound **6d**

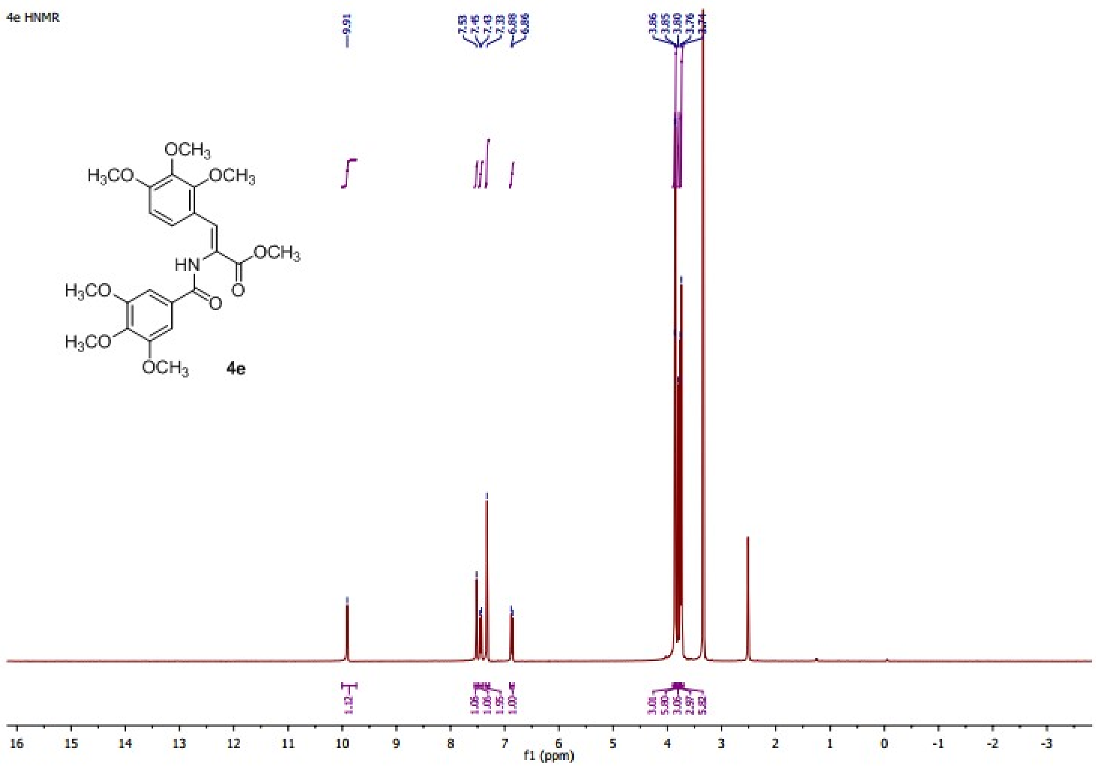

Figure S13: ^{1}H-NMR spectrum of compound **6e**

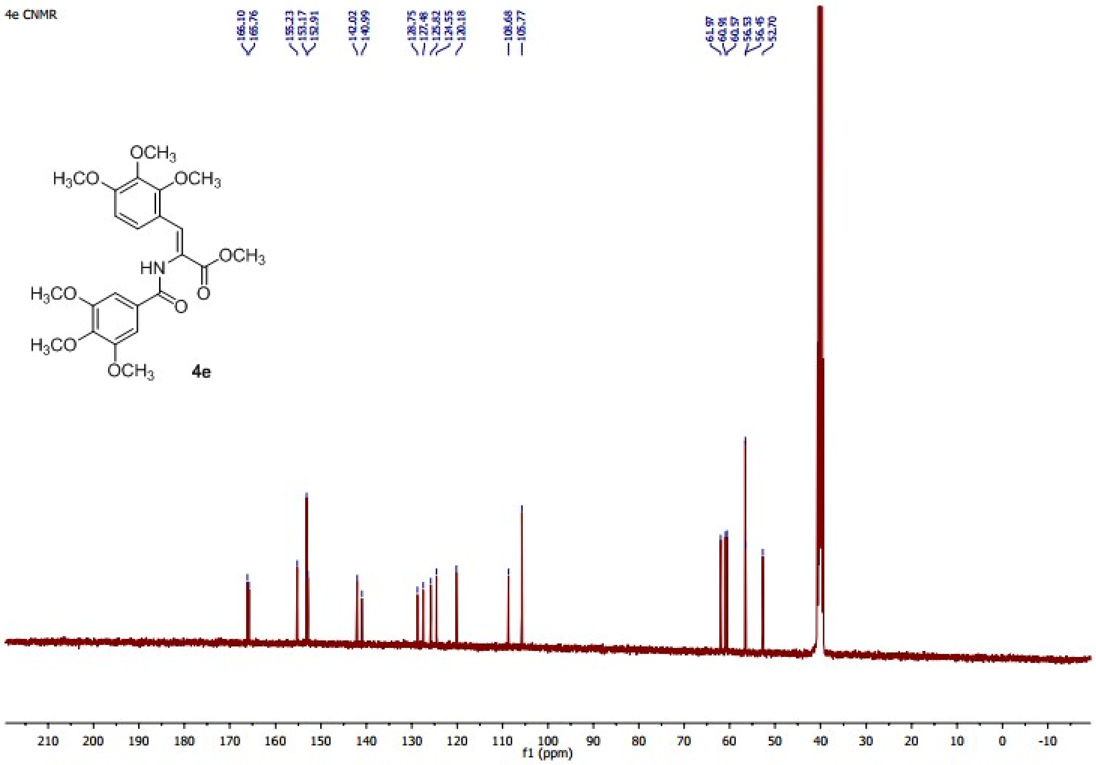

Figure S14: ^{13}C-NMR spectrum of compound **6e**

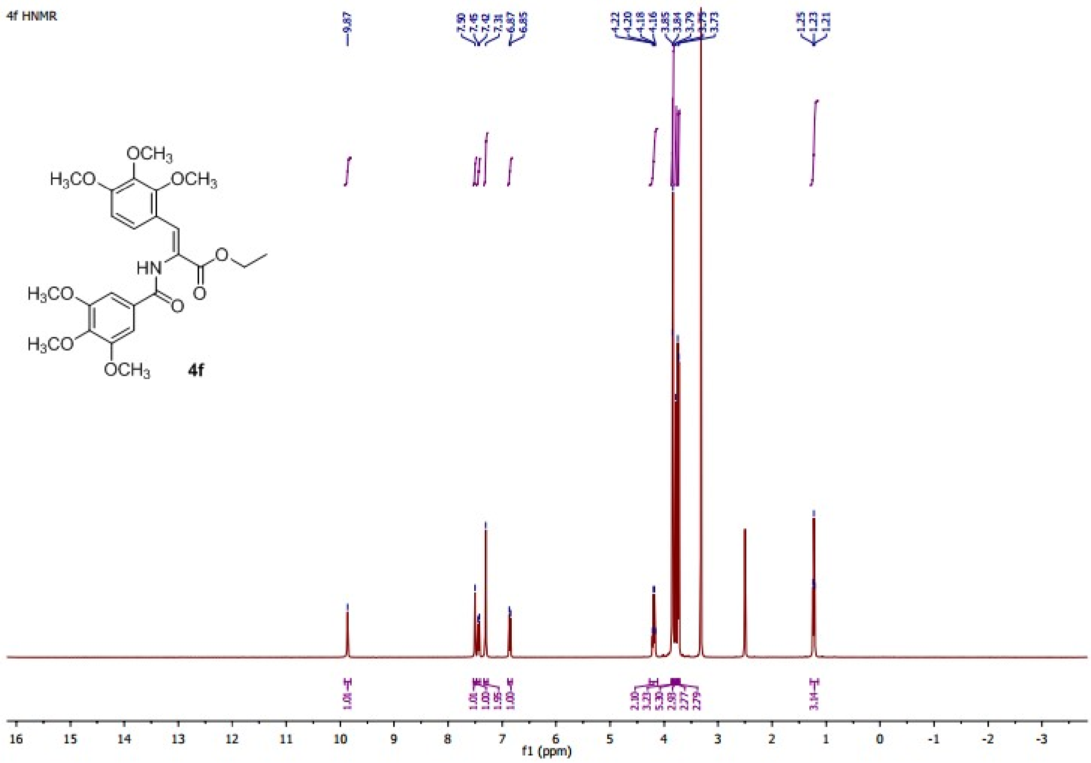

Figure S15: ^{1}H-NMR spectrum of compound **6f**

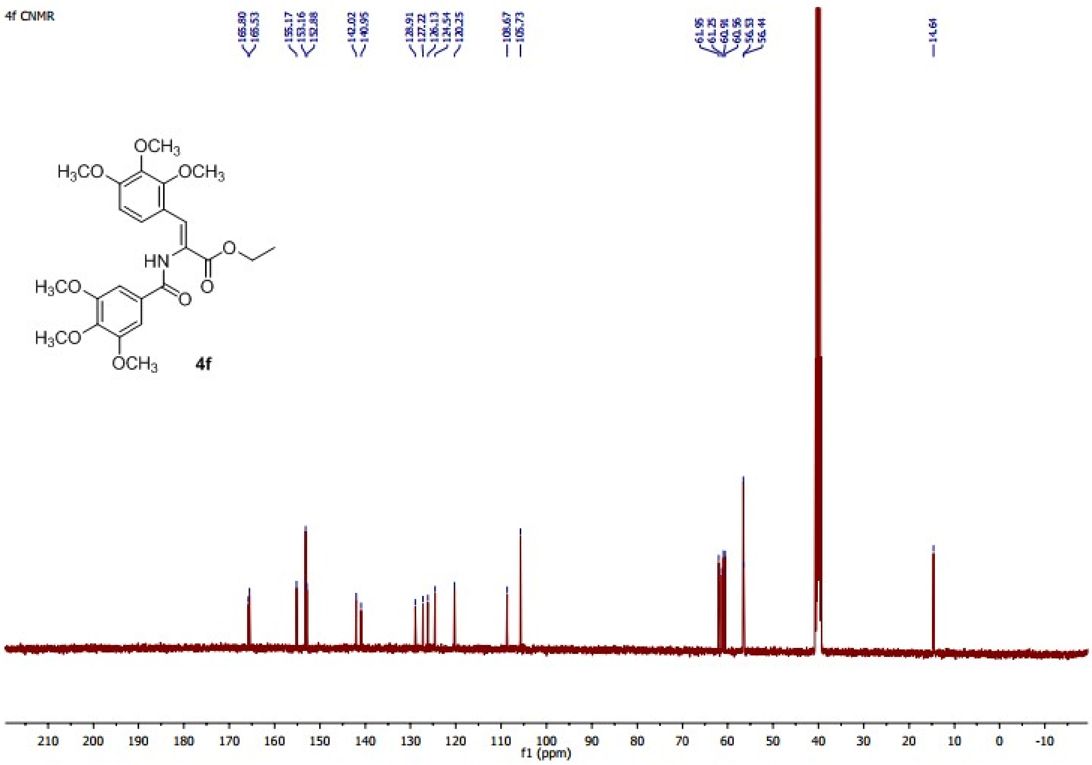

Figure S16: ^{13}C-NMR spectrum of compound **6f**

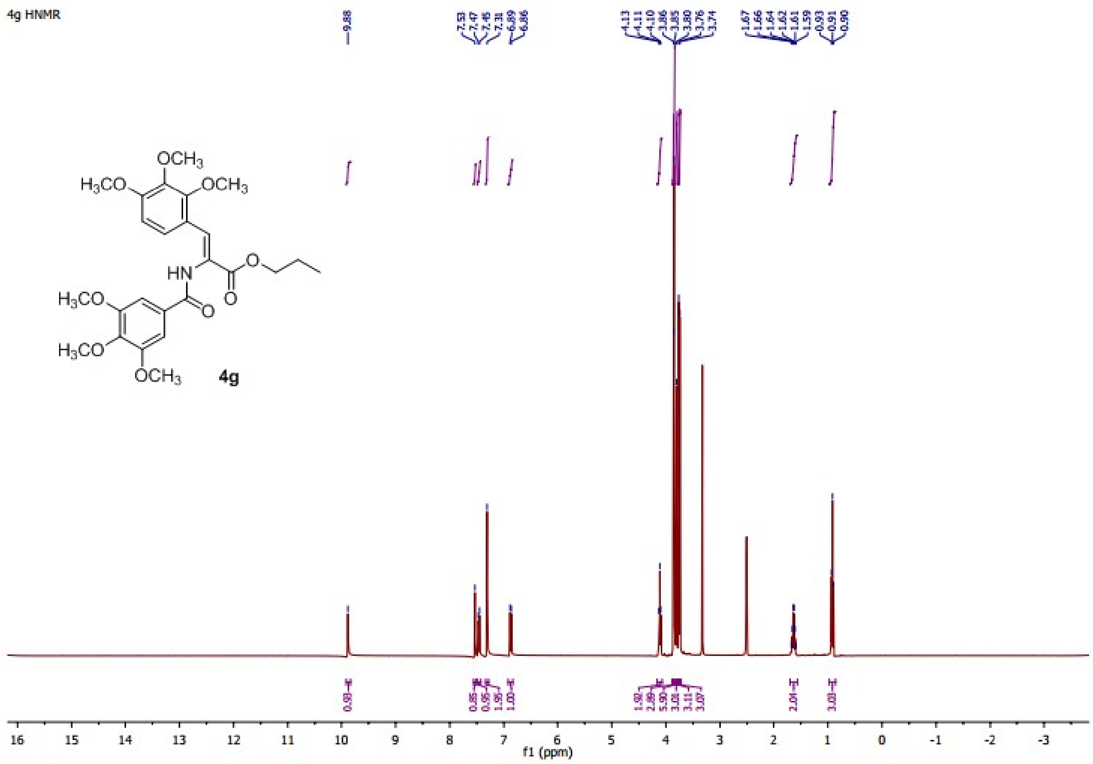

Figure S17: ^{1}H-NMR spectrum of compound **6g**

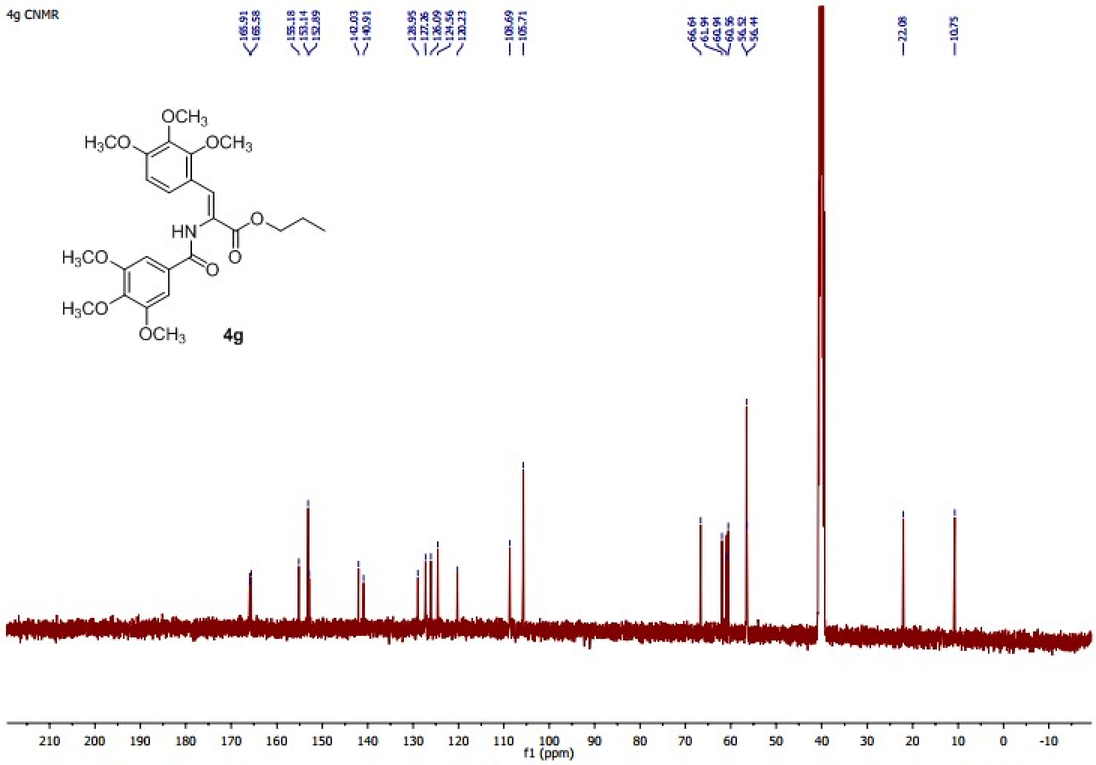

Figure S18: ^{13}C-NMR spectrum of compound **6g**

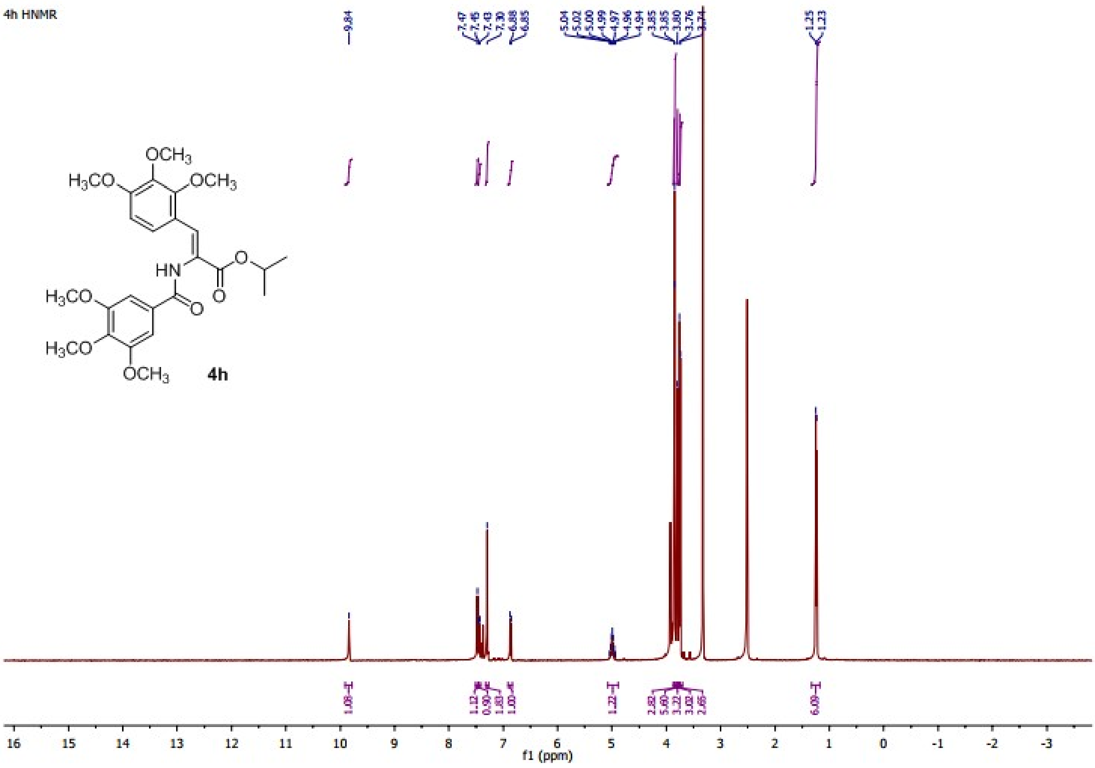

Figure S19: ^{1}H-NMR spectrum of compound **6h**

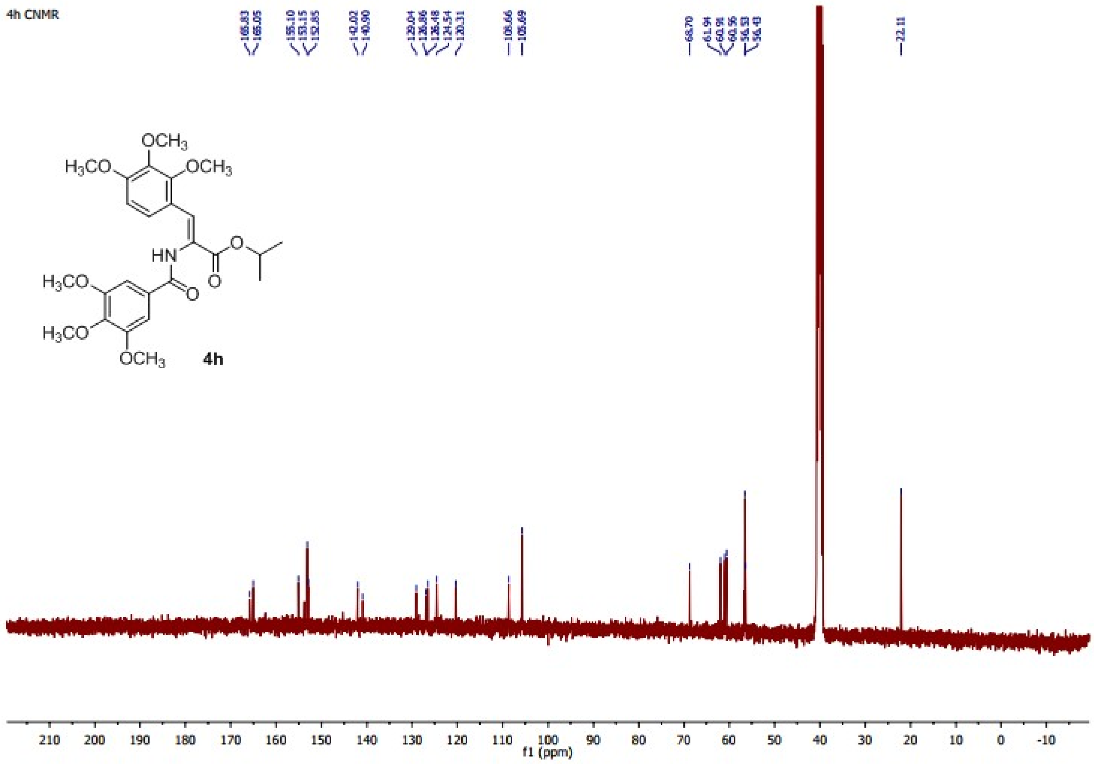

Figure S20: ^{13}C-NMR spectrum of compound **6h**

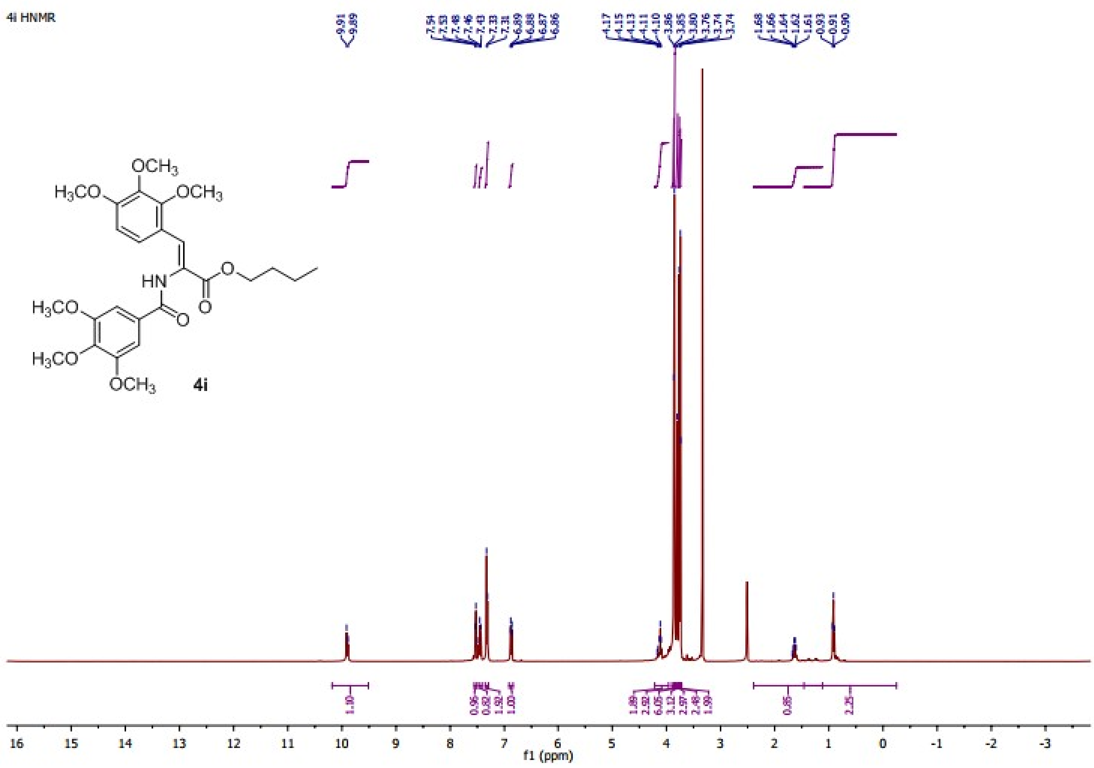

Figure S21: ^{1}H-NMR spectrum of compound **6i**

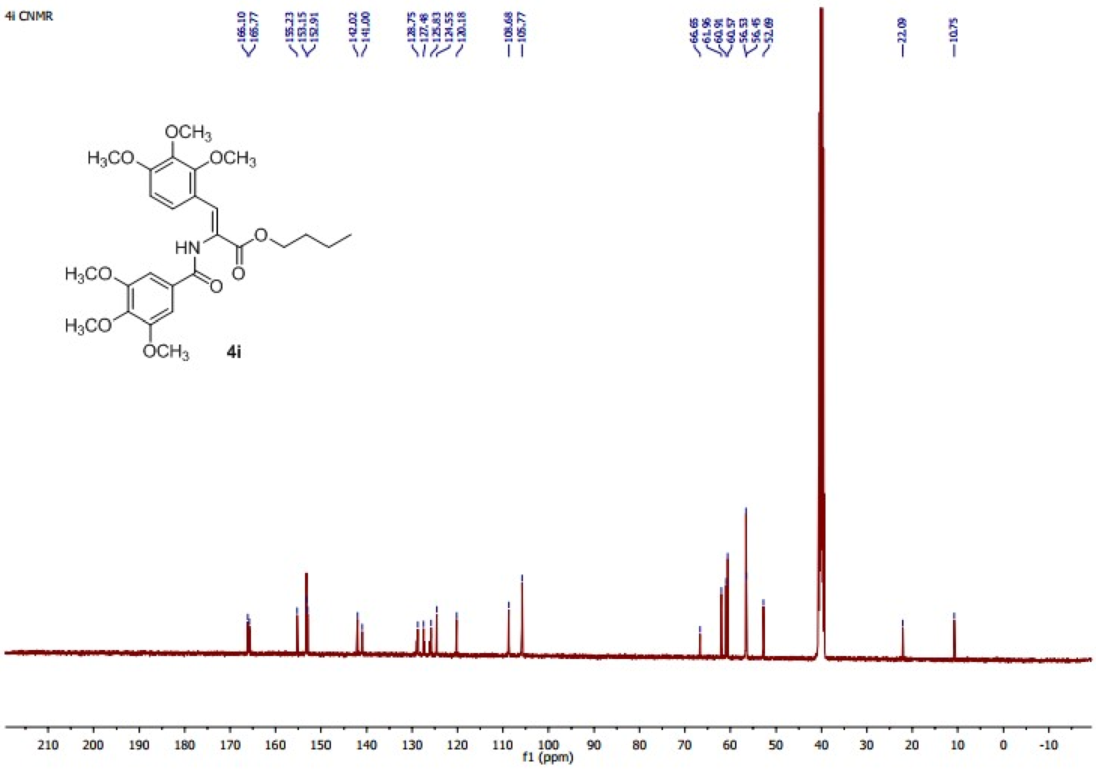

Figure S22: ^{13}C-NMR spectrum of compound **6i**

Appendix A

S4.2. Biological Studies

S4.2.1. Cytotoxic activity evaluation

To measure the cytotoxic actvity of the synthesized acrylate derivatives **5a,b and 6a-i** in breast cancer (MCF-7) cell line. Cell viability assay was assessed using MTT assay method. Cells at density of 1 x 10^4 were seeded in a 96-well plate at 37 ^oC for 24 h under 5% CO_2. After incubation, the cells were treated with different concentrations of the test acrylate derivatives **5a-6i** and incubated for 24 h, then 20 μl of MTT solution at 5 mg/mL was applied and incubated for 4 h at 37 °C. Dimethyl sulphoxide (DMSO) in volume of 100 μl was added to each well to dissolve the purple formazan that had formed. The color intensity of the formazan product, which represents the growth condition of the cells, is quantified by using an ELISA plate reader (EXL 800, USA) at 570 nm absorbance. The experimental conditions were carried out with at least three replicates, and the experiments were repeated at least three times.

S4.2.2. Tubulin polymerization Assay

Compound **6e** and **CA-4** were evaluated for their tubulin inhibitory activity according to manufacturer's instructions using # abcam Human Beta-tubulin simplestep ELISA Kit ab245722.

S4.2.3. Cell cycle analysis of compound 6e

Cell cycle analysis in MCF-7 cells was investigated using fluorescent Annexin V-FITC/ PI detection kit (*BioVision* EZCell^{TM} Cell Cycle Analysis Kit Catalog #K920) by flow cytometry assay. MCF-7 cells at a density of 2 × 10^5 per well were harvested and washed twice in PBS. After that, the cells were incubated at 37 °C and 5% CO_2. The medium was incubated with the tested compound **6e** at ita IC_{50} (μM) for 48 h, washed twice in PBS, fixed with 70% ethanol, rinsed again with PBS. Afterward, medium was stained with DNA fluorochrome PI for 15 min at 37 °C. The samples were immediately analyzed using Facs Calibur flow cytometer (Becton and Dickinson, Heidelberg, Germany).

S4.2.4. Apoptosis assay for compound 6e

Apoptosis in MCF-7 cells was investigated using fluorescent Annexin V-FITC/ PI detection kit (*BioVision* Annexin V-FITC Apoptosis Detection Kit, Catalog #: K101)

by flow cytometry assay. MCF-7 cells at a density of 2 × 10^5 per well were treated with compound **6e** at its IC_{50} (μM) for 48 h, then the cells were harvested and stained with Annexin V-FITC/ PI dye for 15 min in the dark at 37 °C. The samples were immediately analyzed using *FACS Calibur* flow cytometer (Becton and Dickinson, Heidelberg, Germany).

S4.2.5. qRT-PCR measurements of p53, Bax and Bcl2 for compound 6e

Real-time PCR for p53, BAX and Bcl-2 genes expression was done using commercial Qiagen RNA extraction/ BioRad SYBER green PCR master mix according manufacturer's instructions. Briefly, 2 μl of cDNA template, 10 pMol of each forward and reverse primer, 10 μl of 2X Master Mix and to 20 μl total reaction mixture volume by nuclease free water and then was introduced to thermal cycler instrument (Thermo Scientific, USA). The cycling parameters for the PCR amplification were achieved by initial denaturation at 95 °C for 3 minutes followed by 40 cycles of 94 °C for 15 seconds and annealing/extension step at 60 °C for 1 min. Relative quantification of target genes was run on Rotor-Gene 6000 Series Software 1.7 (Build 87).

Table 1: The primer sequences for Real Time PCR assay

Gene	Sequences (5′-3′)
**P53**	F: AGAGTCTATAGGCCCACCCC<br>R: GCTCGACGCTAGGATCTGAC
**Bax**	F: GAGGAACTGGACAGTAACATGGAGCT<br>R: CGGCCCCAGTTGAAGTTGC
**Bcl2**	F: GCCGGTTCAGGTACTCAGTCATC<br>R: GTCACCTTCACCGTTCCA
**GAPDH**	F: GCACCGTCAAGGCTGAGAAC<br>R: ATGGTGGTGAAGACGCCAGT

3050 Spruce Street
Saint Louis, Missouri 63103 USA
Telephone 800-325-5832 • (314) 771-5765
Fax (314) 286-7828
email: techserv@sial.com
sigma-aldrich.com

SIGMA

ProductInformation

IN VITRO TOXICOLOGY ASSAY KIT
MTT BASED
Stock No. TOX-1
Store at 2-8 °C

This kit is designed for determining cell number spectrophotometrically as a function of mitochondrial activity in living cells.

IT IS RECOMMENDED THAT THE ENTIRE PROTOCOL BE REVIEWED BEFORE STARTING THE ASSAY.

PRODUCT DESCRIPTION
Traditionally, the in vitro determination of toxic effects of unknown compounds have been performed by counting viable cells after staining with a vital dye. Alternative methods used are measurement of radioisotope incorporation as a measure of DNA synthesis, counting by automated counters and others which rely on dyes and cellular activity. The MTT system is a means of measuring the activity of living cells via mitochondrial dehydrogenases.

The MTT method is simple, accurate and yields reproducible results. The key component is (3-[4,5-dimethylthiazol-2-yl]-2,5-diphenyl tetrazolium bromide) or MTT. Solutions of MTT, dissolved in medium or balanced salt solutions without phenol red, are yellowish in color. Mitochondrial dehydrogenases of viable cells cleave the tetrazolium ring, yielding purple formazan crystals which are insoluble in aqueous solutions. The crystals are dissolved in acidified isopropanol. The resulting purple solution is spectrophotometrically measured. An increase or decrease in cell number results in a concomitant change in the amount of formazan formed, indicating the degree of cytotoxicity caused by the test material.

[illegible]

KIT COMPONENTS

Catalog No.	Item	Quantity
M-5655	MTT<br>15 mg/vial in serum vial	5
M-8910	MTT Solubilization Solution<br>10% Triton X-100 plus<br>0.1 N HCl in anhydrous<br>isopropanol, 125 ml	1

WARNING: Components in this kit should be carefully handled when using. **MTT** may be harmful if swallowed, inhaled or absorbed through skin. MTT may alter genetic material. **MTT SOLVENT** is flammable and corrosive.

PRODUCT STORAGE
Kit components should be stored at 2-8 °C.

PROCEDURE
The MTT method of monitoring in vitro cytotoxicity is well suited for use with multiwell plates. For best results, cells in the log phase of growth should be employed and final cell number should not exceed 10^6 cells/cm^2. Each test should include a blank containing complete medium without cells.

NOTE: Bacteria, mycoplasma and other microbial contaminants may also cleave the MTT tetrazolium ring. Cultures containing microorganisms should not be assayed using this method.

1. Remove cultures from incubator into laminar flow hood or other sterile work area.
2. Reconstitute each vial of MTT [M-5655] to be [illegible]

Version 1 Last updated 7 April 2020

ab245722
Human Beta-Tubulin
SimpleStep ELISA® Kit

For the quantitative measurement of Beta-Tubulin in human cell and tissue homogenate extract samples.

This product is for research use only and is not intended for diagnostic use.

FOR RESEARCH USE ONLY!

BioVision
BioVision Incorporated

EZCell^{TM} Cell Cycle Analysis Kit

(Catalog #K920-100; 100 assays; Store at -20°C) 10/14

I. Introduction:

Cell cycle is a ubiquitous, complex sequence of events leading to growth and proliferation of cells. Cell Cycle progression is tightly regulated due to its involvement in development, DNA damage and repair, etc. Anomalies in cell cycle progression can lead to tissue hyperplasia and diseases such as cancer. Cell cycle can be subdivided into interphase (G_0/G_1, S and G_2) and mitotic (M) phase (prophase, metaphase, anaphase and telophase). BioVision's Cell Cycle Analysis Kit provides a quick and easy method to detect the number of cells in a cell population, which are at a specific stage of the cell cycle. Our kit utilizes a nuclear dye, the binding of which to nucleic acids in the cell results in fluorescence signal, which is proportional to cellular DNA content. The percentages of cells in different phases of the cell cycle (G_0/G_1, S, and G_2/M) can be quantified by flow cytometry. Our method is non-radioactive, rapid and accurate and can be used for high throughput cell cycle analysis with contemporary flow cytometer instruments.

II. Application:

- Analysis of cell cycle regulation in response to growth factors, cytokines, mitogens, and nutrients, etc.
- Monitoring of cell cycle progression
- Study effects of drugs which affect cell growth and division

III. Sample Type:

- Adherent or Suspension cells

IV. Kit Contents:

Components	K920-100	Cap Code	Part Number
10X Cell Cycle Assay Buffer	50 ml	NM	K920-100-1
Enzyme A Solution	2 x 250 µl	Blue	K920-100-2
Nuclear Dye	2 x 1 ml	Red	K920-100-3

V. User Supplied Reagents and Equipment:

- 6-well tissue culture plate
- Cell Culture Media and Fetal Bovine Serum
- 70% Ethanol (Pre-chilled on ice)
- Flow Cytometer with excitation filter at 488 nm wavelength

VI. Reagent Preparation and Storage:

Store the kit at -20°C, protected from light. Warm all reagents to room temperature (RT) before use. Read the entire protocol before performing the assay.

- **10X Cell Cycle Assay Buffer:** Dilute10X Cell Cycle Assay Buffer with ddH_2O to prepare 1X working solution. Pre-chill 1X Cell Cycle Assay Buffer on ice before use.
- **Nuclear Dye:** For long-term storage, aliquot and store at -20°C to avoid repeated freeze/thaw.
- **Staining Solution:** Before performing the analysis, prepare Staining Solution - for every 20 samples (based on 6-well plate sample size): add 100 µl of Enzyme A Solution and 400 µl of Nuclear Dye into 10 ml of 1X Cell Cycle Assay Buffer, mix well and protect from light. Stable for one week at 4°C.

VII. Cell Cycle Analysis Protocol:

1. **Sample Preparation:** Grow cells of interest (2-5 x 10^5 cells/well) in desired medium and culture conditions preferably in 6-well plates for 24 hr prior to the experiment. Synchronize cells with culture medium containing 0.1% FBS for 24 hr. Treat cells with test compounds in culture medium containing 10% FBS for 4-24 hr. As controls, incubate cells of interest in culture medium with 10% FBS without any test compound. Harvest cells and centrifuge at 400 x g for 5 min. Remove the supernatant and wash cells in 2 ml ice cold 1X Cell Cycle Assay Buffer, centrifuge cells at 400 x g for 5 min., remove the supernatant and save the cell pellet.

   Notes:

   a. Cell density depends on the cell type, and it may be necessary to adjust the cell numbers for optimal cell density.

   b. For longer incubation times, change culture medium containing 10 % FBS with test compounds every 24 hr.

BIO-RAD

iScript™ One-Step RT-PCR Kit with SYBR® Green

50 x 50 µl reactions	170-8892
200 x 50 µl reactions	170-8893

For Research purposes only
Store at -20°C, protect from light

The iScript One-Step RT-PCR Kit with SYBR® Green is a convenient and highly sensitive solution for real-time quantitative PCR of RNA templates. cDNA synthesis and PCR amplification are carried out in the same tube. This kit is optimized to deliver maximum RT-PCR efficiency, sensitivity and specificity without compromising fluorescent signal. The proprietary reaction buffer has been specifically formulated to maximize activities of both the reverse transcriptase and the iTaq™ DNA polymerase, while minimizing the potential for primer-dimer and other non-specific PCR artifacts.

The 2X SYBR® Green RT-PCR Reaction Mix includes the iTaq antibody-mediated hot-start DNA polymerase that sequesters activity prior to the initial PCR denaturation step. Upon heat activation, the antibody denatures irreversibly, releasing fully active and unmodified iTaq DNA polymerase. Highly specific amplification is essential for successful qRT-PCR with SYBR® Green I technology, since this dye binds to any double-stranded DNA generated during amplification.

Storage and Stability

Store the iScript SYBR® Green RT-PCR Kit at -20°C in a constant temperature freezer, protected from light. When stored under these conditions, the kit components are stable for a minimum of one year after ship date. For extended stability, the iScript Reverse Transcriptase for One-Step RT-PCR may be stored at -70°C. Repeated freeze-thaw cycles should be avoided. For convenience, the 2X SYBR® Green RT-PCR Reaction Mix may be stored unfrozen at 2°C to 8°C for up to 4 months.

Kit Contents

**Reagent**	**Description**
iScript Reverse Transcriptase for One-Step RT-PCR (yellow cap)	Optimized 50X formulation of iScript MMLV reverse transcriptase for One-Step RT-PCR procedures
2X SYBR® Green RT-PCR Reaction Mix (green cap)	2X reaction buffer containing 0.4 mM of each dNTP (dATP, dCTP, dGTP, dTTP), magnesium chloride, iTaq DNA polymerase, 20 nM fluorescein, SYBR® Green I dye, stabilizers
Nuclease-free H_2O	

BioVision

rev. 04/11

For research use only

Annexin V-FITC Apoptosis Detection Kit

(Catalog #: K101-25, -100, -400; Store at 4°C; Stable for one year)

I. Introduction:

Annexin V Apoptosis Detection Kit is based on the observation that soon after initiating apoptosis, cells translocate the membrane phosphatidylserine (PS) from the inner face of the plasma membrane to the cell surface. Once on the cell surface, PS can be easily detected by staining with a fluorescent conjugate of Annexin V, a protein that has a high affinity for PS. The one-step staining procedure takes only 10 minutes. Detection can be analyzed by flow cytometry or by fluorescence microscopy. The kit can differentiate between apoptosis and necrosis when performing both Annexin V-FITC and PI staining.

II. Kit Contents:

Components	K101-25	K101-100	K101-400	Part Number
	25 assays	100 assays	400 assays	
Annexin V-FITC	125 µl	500 µl	2 ml	K101-XX(X)-1
1X Binding Buffer	12.5 ml	50 ml	2 x 100 ml	K101-XX(X)-2
Propidium Iodide (PI)	125 µl	500 µl	2 ml	K101-XX(X)-3

III. Annexin V-FITC Assay Protocol:

A. Incubation of cells with Annexin V-FITC

1. Induce apoptosis by desired method.
2. Collect 1-5 x 10^5 cells by centrifugation.
3. Resuspend cells in 500 µl of 1X Binding Buffer.
4. Add 5 µl of Annexin V-FITC and 5 µl of propidium iodide (PI 50µg/ml, optional.)
5. Incubate at room temperature for 5 min in the dark.
   Proceed to B or C below depending on method of analysis.

B. Quantification by Flow Cytometry

Analyze Annexin V-FITC binding by flow cytometry (Ex = 488 nm; Em = 530 nm) using FITC signal detector (usually FL1) and PI staining by the phycoerythrin emission signal detector (usually FL2).

For adherent cells, gently trypsinize and wash cells once with serum-containing media before incubation with Annexin V-FITC (A.3-5).

C. Detection by Fluorescence Microscopy

1. Place the cell suspension from Step A.5 on a glass slide. Cover the cells with a glass coverslip.

For analyzing adherent cells, grow cells directly on a coverslip. Following incubation (A.5), invert coverslip on a glass slide and visualize cells. The cells can also be washed and fixed in 2% formaldehyde before visualization. (Cells must be incubated with Annexin V-FITC before fixation since any cell membrane disruption can cause nonspecific binding of Annexin V to PS on the inner surface of the cell membrane.)

2. Observe the cells under a fluorescence microscope using a dual filter set for FITC & rhodamine.

Cells that have bound Annexin V-FITC will show green staining in the plasma membrane. Cells that have lost membrane integrity will show red staining (PI) throughout the nucleus and a halo of green staining (FITC) on the cell surface (plasma membrane).

RELATED PRODUCTS:

Apoptosis Detection Kits & Reagents
- Annexin V Kits & Bulk Reagents
- Caspase Assay Kits & Reagents
- Mitochondrial Apoptosis Kits & Reagents
- Nuclear Apoptosis Kits & Reagents
- Apoptosis Inducers and Set
- Apoptosis siRNA Vectors

Cell Fractionation System
- Mitochondria/Cytosol Fractionation Kit
- Nuclear/Cytosol Fractionation Kit
- Membrane Protein Extraction Kit
- Cytosol/Particulate Rapid Separation Kit
- Mammalian Cell Extraction Kit
- FractionPREP Fractionation System

Cell Proliferation & Senescence
- Quick Cell Proliferation Assay Kit
- Senescence Detection Kit
- High Throughput Apoptosis/Cell Viability Assay Kits
- LDH-Cytotoxicity Assay Kit
- Bioluminescence Cytotoxicity Assay Kit
- Live/Dead Cell Staining Kit

Cell Damage & Repair
- HDAC & HAT Fluorometric & Colorimetric Assays & Drug Discovery Kits
- DNA Damage Quantification Kit
- Glutathione & Nitric Oxide Fluorometric & Colorimetric Assay Kits

Signal Transduction
- cAMP & cGMP Assay Kits
- Akt & JNK Activity Assay Kits
- Beta-Secretase Activity Assay Kit

Adipocyte & Lipid Transfer
- Recombinant Adiponectin, Survivin, & Leptin
- CETP & PLTP Activity Assay & Drug Discovery Kits
- Total Cholesterol Quantification Kit

Molecular Biology & Reporter Assays
- siRNA Vectors
- Cloning Insert Quick Screening Kit
- Mitochondrial & Genomic DNA Isolation Kits
- 5 Minutes DNA Ligation Kit
- 20 Minutes Gel Staining/Destaining Kit
- β-Galactosidase Staining Kit & Luciferase Reporter Assay Kit

Growth Factors and Cytokines

Page 1

BioVisionResearch Products
980 Linda Vista Avenue, Mountain View, CA 94043 USA

Tel: 650-428-0236 • Fax: 650-428-0336 • www.biovision.com
Email: tech@biovision.com
